# Efficacy of Lapatinib in Therapy-Resistant HER2-Positive Circulating Tumor Cells in Metastatic Breast Cancer

**DOI:** 10.1371/journal.pone.0123683

**Published:** 2015-06-17

**Authors:** Sofia Agelaki, Antonia Kalykaki, Harris Markomanolaki, Maria A. Papadaki, Galatea Kallergi, Dora Hatzidaki, Kostas Kalbakis, Dimitrios Mavroudis, Vassilis Georgoulias

**Affiliations:** 1 Department of Medical Oncology, University Hospital of Heraklion, Heraklion, Crete, Greece; 2 Laboratory of Tumor Biology, School of Medicine, University of Crete, Heraklion, Crete, Greece; The First Affiliated Hospital with Nanjing Medical University, CHINA

## Abstract

**Background:**

To evaluate the efficacy of lapatinib, a dual EGFR and HER2 tyrosine kinase inhibitor, in therapy-resistant HER2-positive CTCs in metastatic breast cancer (MBC).

**Patients and Methods:**

Patients with MBC and HER2-positive CTCs despite disease stabilization or response to prior therapy, received lapatinib 1500 mg daily in monthly cycles, till disease progression or CTC increase. CTC monitoring was performed by immunofluorescent microscopy using cytospins of peripheral blood mononuclear cells (PBMCs) double stained for HER2 or EGFR and cytokeratin.

**Results:**

A total of 120 cycles were administered in 22 patients; median age was 62.5 years, 15 (68.2%) patients were post-menopausal and 20 (90.1%) had HER2-negative primary tumors. At the end of the second course, HER2-positive CTC counts decreased in 76.2% of patients; the median number of HER2-positive CTCs/patient also declined significantly (p = 0.013), however the decrease was significant only among patients presenting disease stabilization (p = 0.018) but not among those with disease progression during lapatinib treatment. No objective responses were observed. All CTC-positive patients harbored EGFR-positive CTCs on progression compared to 62.5% at baseline (p = 0.054). The ratio of EGFR-positive CTCs/total CTCs detected in all patients increased from 17.1% at baseline to 37.6% on progression, whereas the mean percentage of HER2-negative CTCs/patient increased from 2.4% to 30.6% (p = 0.03).

**Conclusions:**

The above results indicate that lapatinib is effective in decreasing HER2-positive CTCs in patients with MBC irrespectively of the HER2 status of the primary tumor and imply the feasibility of monitoring the molecular changes on CTCs during treatment with targeted agents.

**Trial Registration:**

Clinical trial.gov NCT00694252

## Introduction

Several lines of evidence suggest that metastatic spread in breast cancer is the result of tumour cell dissemination from the primary site to distant organs which occurs early in the course of malignant progression [1]. Indeed, cytokeratin-positive epithelial cells in the bone marrow aspirates (disseminated tumor cells; DTCs) and the peripheral blood (circulating tumor cells; CTCs) have been identified in otherwise metastasis-free patients with early breast cancer by the use of immunocytochemistry or Reverse Transcription Polymerase Chain Reaction (RT-PCR) [2]; the detection of these cells has been proven to be an independent unfavourable prognostic factor associated with increased distant relapse rate and decreased overall survival [3–6]. In addition, CTCs can be detected in 40–70% of patients with metastatic breast cancer (MBC) [7,8]. Using the CellSearch platform, the presence of 5 or more CTCs/7.5 ml of peripheral blood in women with MBC before starting a new line of treatment has been shown to predict progression-free and overall survival, whereas CTC counts in the first follow-up visit were also predictive of patient outcome [9].

Besides CTC detection, significant effort has been undertaken towards CTC characterization [10]. Thus it has been recognized that CTCs consist of a heterogeneous population of cells [11]. In addition, a significant discordance has been observed between the molecular characteristics of the primary tumor and those of corresponding CTCs [12–15]. Accordingly, taking into account the ease of blood sampling, it has been suggested that CTCs could serve as a “real-time liquid biopsy” for the identification of relevant therapeutic targets [16]. Our group, using a double staining immunofluorescence assay, has previously reported the presence of HER2- and EGFR-expressing CTCs in the peripheral blood of MBC patients [17,18]. The downstream PI3K/Akt signalling pathway was also shown to be activated in CTCs suggesting the involvement of this pathway in CTC survival [18].

Disease relapse after prior therapy is the major cause of mortality in patients with metastatic disease. Residual tumor cells that survive previous treatments could be responsible for this relapse. Indeed it has been shown that CTCs are detected after the administration of chemotherapy and/or hormonal therapy and their persistence is associated with poor clinical outcome [19–21]. Targeting these CTCs could be beneficial for patients with breast cancer. The primary objective of this pilot study was to assess the efficacy of the dual HER2 and EGFR inhibitor, lapatinib, on chemotherapy- or/and hormonal therapy-resistant CTCs in patients with MBC.

## Patients and Methods

The protocol for this trial and supporting CONSORT checklist are available as supporting information; see S1 CONSORT Checklist and S1 Protocol.

### Patients

Women with MBC and at least one HER2-positive CTC per 10^6^ PBMCs irrespectively of the HER2 status of the primary tumor, were eligible. Patients had to have non-progressive disease (stable disease or partial response) after the completion of prior therapy for advanced disease. Other eligibility criteria were: age >18 years old, adequate bone marrow, renal and liver function, Left Ventricular Ejection Fraction (LVEF) within normal range and performance status 0–2 (World Health Organization, WHO); at least 4 weeks to have elapsed after the end of prior treatment. Before treatment initiation, all patients had a complete physical examination, blood chemistry and a diagnostic evaluation including computed tomography scans (CT scans) of the chest and abdomen [or magnetic resonance imaging (MRI)] if clinically indicated as well as whole body radionuclide bone scan. Physical examination and blood chemistry were repeated every month. Patients were assessed by bone scan, CT scans (or MRI if indicated) every 3 months (or at any time in case of suspected clinical disease progression) and response evaluation was performed according to Response Evaluation Criteria In Solid Tumors (RECIST), version 1.0 [22]. No other anticancer treatment was allowed during the period of lapatinib administration. All patients provided written informed consent in order to participate in the trial. The study protocol was approved by the Ethics and Scientific Committees of the University Hospital of Heraklion as well as by the Hellenic Drug Organization (EOF). Clinical investigation has been conducted according to the principles expressed in the Declaration of Helsinki.

### Treatment administration

Patients received lapatinib, 1500 mg/day orally in monthly cycles. CTC counts were evaluated at baseline and then monthly prior to the initiation of each subsequent cycle of treatment. Patients presenting a CTC response before the start of the second cycle (CTC counts lower than the baseline values), or with stable CTC counts were continued on lapatinib. Those presenting an increase in CTC counts (CTC counts higher than the baseline values) were taken off study providing that further increase was documented before the third course. In subsequent cycles, treatment was also discontinued in case of two consecutive increases in CTC counts. Moreover, treatment was discontinued in case of disease progression, occurrence of unacceptable toxicity or consent withdrawal, irrespectively of CTC counts.

### Sample collection

Peripheral blood (10 ml in EDTA) was obtained before the initiation of lapatinib and monthly thereafter. To avoid cell contamination from the epidermis the first 5ml of blood were discarded. Blood was diluted with equal volume of 0.9% NaCl and peripheral blood mononuclear cells (PBMCs) were obtained by gradient density centrifugation using Ficoll Hypaque-1077 (Sigma Chemical Company, St. Louis, MO) at 1800rpm for 30 min at room temperature. The interface cells were removed, washed twice with 40 mL of sterile PBS (pH: 7.3) at 1500 rpm for 10 min, and resuspended in 10mL of PBS. PBMCs cytospins were prepared using aliquots of 500,000 cells by cyto-centrifugation at 2000 rpm for 2 min and slides were dried up and stored at -80 ^0^C until further use.

### Double immunofluorescence for the detection of HER2 and EGFR on PBMC cytospins

The presence of HER2-positive CTCs in cytospin preparations of PBMCs was determined using the mouse A45-B/B3 (detecting CK8, CK18, and CK19; Micromet, Munich, Germany) antibody and the rabbit HER2 antibody (Cell Signaling Technology) as previously described [18]. Briefly, PBMCs’ cytospins were fixed with cold aceton:methanol 9:1 for 20 minutes and stained with A45-B/B3 antibody for 1h. Subsequently, the same slide was stained with anti-HER2 for 1h. For the detection of EGFR positive CTCs, staining was performed using the EGFR antibody (Santa Cruz, Santa Cruz, CA, USA) for 1h [18]. Cells were then incubated with the corresponding secondary antibodies for 45 minutes. To verify the specificity of the staining procedure, positive and negative controls for both markers were included in each experiment. HER2 amplified SKBR3 breast cancer cells that express both HER2 and EGFR were used as positive controls [18]; negative controls were also prepared using SKBR3 cells by omitting HER2 or EGFR primary and adding the secondary immunoglobulin G (IgG) isotype antibody [18]. Cytospins of PBMCs obtained from 10 female healthy blood donors were also double stained with A45-B/B3 and HER2 or EGFR antibodies.

The cytomorphological criteria proposed by Meng et al [23] (high nuclear/cytoplasmic ratio, larger cells than white blood cells, etc) were used to characterize a CK-positive cell as a CTC. CTCs were characterized as HER2-positive if the intensity of HER2-staining was higher compared to the HER2-negative control. Similarly, a CTC was determined as EGFR-positive if the intensity of EGFR staining was higher than the respective negative control. A total of 10^6^ PBMCs per patient were analyzed by the use of immunofluorescence microscopy (Leica DM 2500). Patients with at least one HER2-positive or EGFR-positive CTC per 10^6^ PBMCs were determined as HER2-positive or EGFR-positive, respectively. The evaluation of PBMC cytospins from healthy female blood donors revealed that there were no cells staining positive for HER2 or EGFR and cytokeratins (CK) (data not shown). Results are expressed as number of CTCs/10^6^ PBMCs.

### Study design

This is a pilot feasibility study designed to evaluate the effect of lapatinib on HER2-positive CTC counts in patients with MBC who present disease stabilization or response after the completion of prior therapy for metastatic disease. Secondary endpoints were to correlate the kinetics of HER2-positive CTCs with response to treatment and the assessment of treatment-induced toxicity. As a correlative study, EGFR expression on CTCs at baseline and on progression was also evaluated.

### Statistical analysis

This is a pilot study aiming to demonstrate the effect of lapatinib on HER2-positive CTC counts. Since no prior experience exists for this approach, no formal sample size estimation was carried out.

Progression-free survival (PFS) was calculated from the date of the study enrolment until the date of the first evidence of disease and/or CTC progression. The Kaplan–Meier method was used to plot the corresponding PFS curve. The distribution of values was tested by Kolmogorov-Smirnov normality test. Categorical variables were compared by Chi-square test, whereas Wilcoxon signed rank test (2 related samples) was used to compare quantitative factors between different time points. Statistical analyses were performed using IBM SPSS Statistics version 20. P values were considered statistically significant at the 0.05 level.

## Results

### Patients

**Fig 1:**
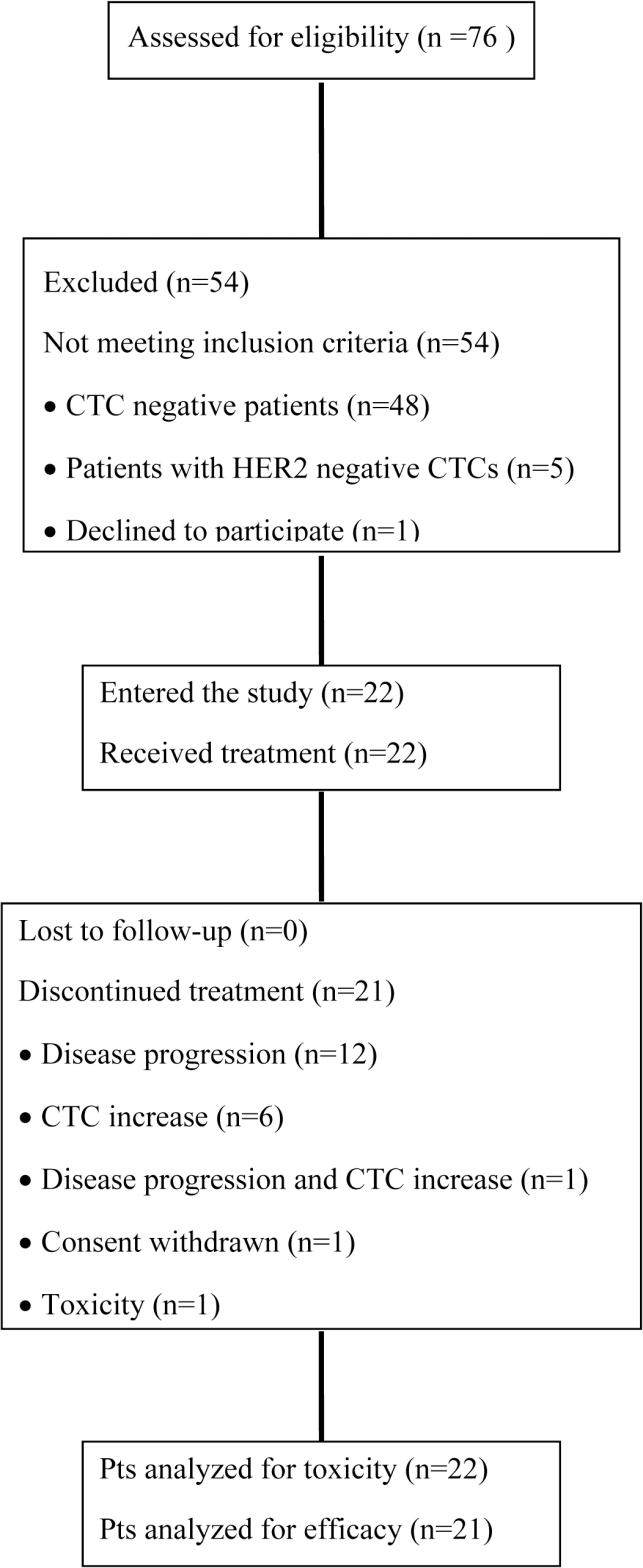
CONSORT flow diagram of the study.

Seventy-six consecutive patients with MBC and disease stabilization or response following previous treatment were screened from September 2008 to November 2011. Twenty-eight (36.9%) were CTC-positive, 23 (30.3%) had at least one HER2-positive CTC and 22 patients were enrolled into the study. The last patient was enrolled on November 2011 and the date of the last follow-up was on February 2013. All patients were evaluable for toxicity and 21 for efficacy analysis. A CONSORT flow diagram of the study is presented in Fig 1. Patients’ characteristics are listed in Table 1. Median age was 62.5 years, 15 (68.2%) patients were post-menopausal and 20 (90.1%) had HER2-negative primary tumors. Twelve (54.5%) patients had visceral disease and 11 (50%) had received 2 or more prior lines of treatment for advanced disease.

**Table 1:** Patient characteristics.

	Patients	
	n = 22	%
**Age**		
Median (min-max)	62.5 (37–77)	
**Menopausal status**		
Pre-menopausal	7	31.8
Post-menopausal	15	68.2
**HER2 status of primary tumor**		
Positive	2	9.1
Negative	20	90.1
**Hormone receptor status**		
ER positive PR positive	14	63.6
ER positive PR negative	4	18.2
ER negative PR positive	2	9.1
Uknown	2	9.1
**Disease sites**		
Visceral	12	54.5
Non-visceral	10	45.5
**Number of prior regimens**		
1	11	50.0
≥2	11	50.0
**Prior hormone therapy in the metastatic setting**		
Yes	20	90.9
No	2	9.1

### Effect of lapatinib on HER2-positive CTCs

**Fig 2:**
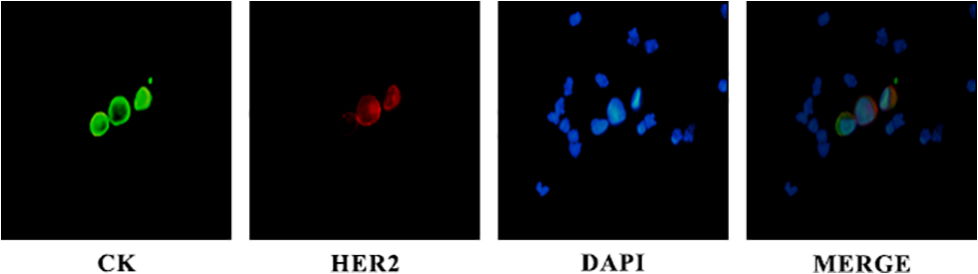
A representative picture of CTCs detected on a patient’s cytospin. Cells were double stained using A45 B/B3 (green), HER2 (red) antibodies, and DAPI (blue) and evaluated by immunofluorescence microscopy. Presented is a HER2-negative CTC in comparison with two HER2-positive CTCs.

A median of 130 HER2-positive CTCs (range 1–617) per patient were detected at baseline among the 21 evaluable patients; HER2 expression (Fig 2) was evident in 93.45% of the total number of CTCs detected in these patients.

**Fig 3:**
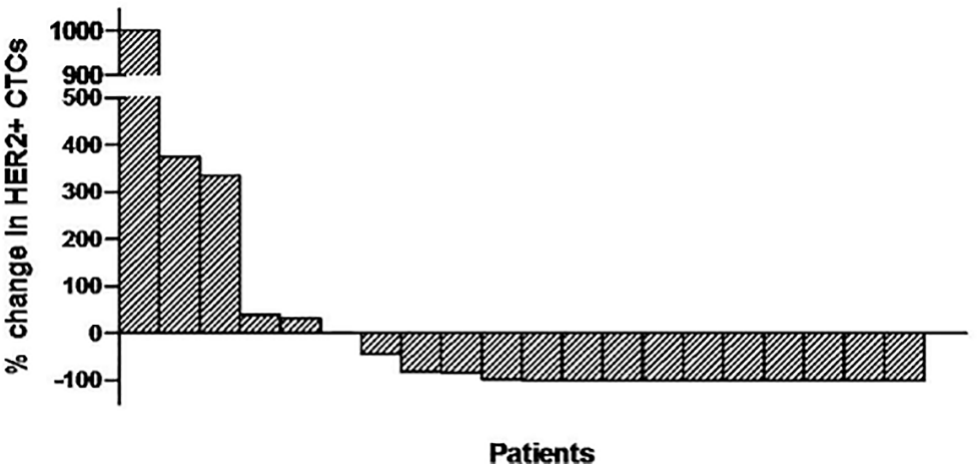
Waterfall plot of the % change in HER2-positive CTC numbers in patients treated with lapatinib (n = 20) after the completion of the first treatment cycle as compared to the baseline; in one patient no information on CTC counts was available.

**Fig 4:**
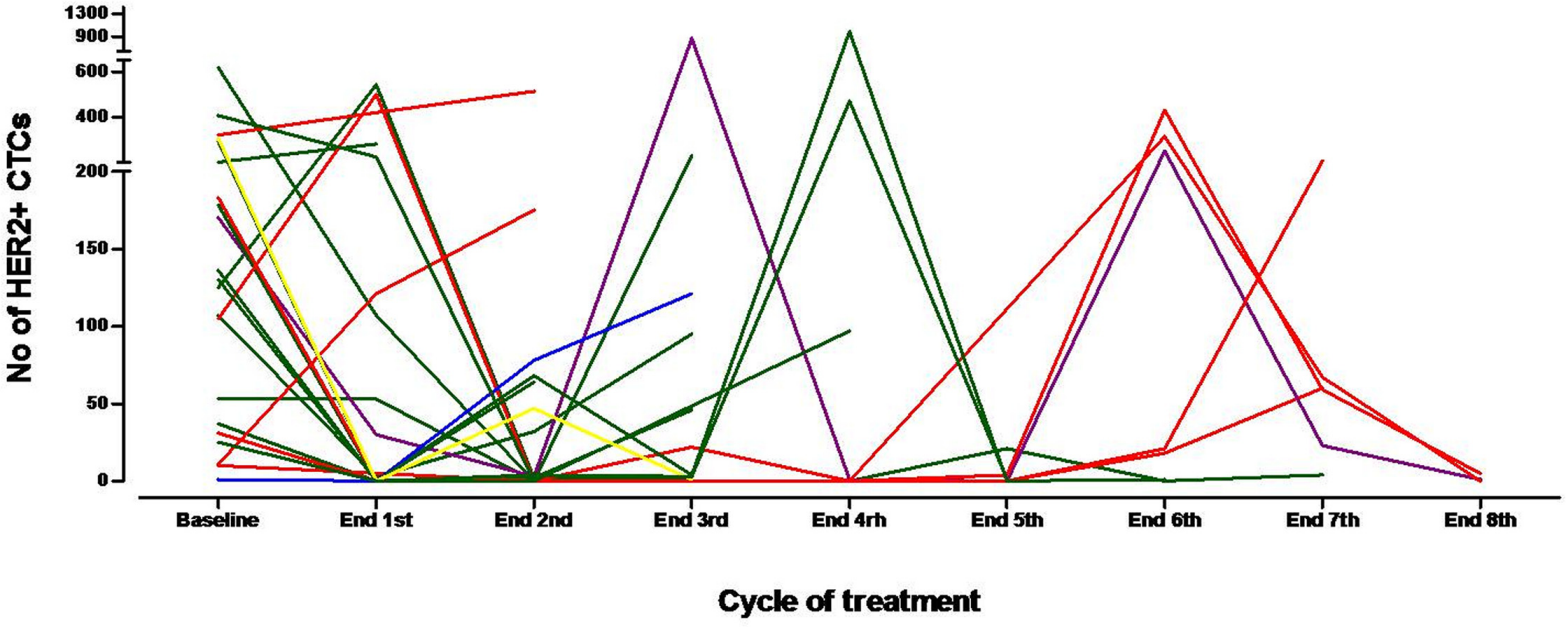
Patient-specific trajectories of HER2-positive CTCs during the first 8 cycles of treatment. The trajectories of CTCs are marked red in patients that discontinued treatment due to increase in CTC counts (n = 6), green in cases with disease progression (n = 12) and blue for both CTC increase and disease progression (n = 1). One patient withdrew consent (depicted in yellow) and one was still on treatment at the time of the analysis (purple).

At the end of the first treatment cycle, a decrease in HER2-positive CTC counts was observed in 14 (66.6%) patients (95% C.I: 49.9%-90.1%); the median percentage of decrease per patient was 100% (range, 45–100%). In 5 (23.8%) patients, HER2-positive CTC counts increased and in one (4.8%) remained stable (Fig 3). In one (4.8%) patient although blood sample was not available for CTC analysis after the end of the first cycle, treatment with lapatinib was continued due to lack of clinical disease progression. At the end of the second treatment cycle, HER2-positive CTCs declined in 16 (76.2%) of 21 patients (95% C.I: 58.0%-94.4%). The median time to nadir counts was 1 month (range, 1–5 months). Total CTC and HER2-positive CTC counts during treatment in all patients are presented in Table 2. Furthermore, Fig 4 depicts the patient-specific trajectories of HER2-positive CTCs.

**Table 2:** Total CTCs and HER2-positive CTC counts during lapatinib treatment and patient outcome.

Patient no	Baseline		Post-1st		Post-2nd		Post-3rd		Post-4th		Post-5th		Post-6th		Post-7th		Post-8th		Best response(cycles on Tx)
	CTC counts																		
	Total	HER2	Total	HER2	Total	HER2	Total	HER2	Total	HER2	Total	HER2	Total	HER2	Total	HER2	Total	HER2	
**1**	133	125	551	542	2	2	46	46											PD (3)
**2**	201	201	281	281															PD (1)
**3**	107	107	3	3	33	32	95	95											PD (3)
**4**	37	37	0	0	MV^a^	MV													PD (2)
**5**	53	53	53	53	0	0	0	0	0	0	21	21	0	0	4	4			SD (7)
**6**	617	617	107	107	0	0	230	230											PD (3)
**7**	31	31	0	0	0	0	0	0	0	0	0	0	21	18	60	60			SD (7)
**8**	170	170	30	30	5	3	872	872	1	1	0	0	252	250	23	23	1	1	SD (27, ongoing)
**9**	178	178	0	0	2	2	1	1											PD (3)
**10**	10	10	MV	MV	0	0	0	0	0	0	MV	MV	314	314	67	67	0	0	SD (10)
**11**	105	105	509	500	0	0	MV	MV	0	0	0	0	21	21	206	206			SD (7)
**12**	183	183	1	1	0	0	22	22	0	0	4	4	447	431	127	59	5	5	SD (15)
**13**	292	292	0	0	68	68	4	4	991	991	0	0	1	1					SD (6)
**14**	1	1	0	0	78	78	122	121											PD (3)
**15**	130	130	0	0	64	64													PD (2)
**16**	11	11	121	121	175	175													SD (2)
**17**	321	321	420	420	513	513													PD (2)
**18**	406	406	398	223	0	0	MV	MV											PD (3)
**19**	160	136	0	0	0	0	MV	MV	107	97	MV	MV							SD (5)
**20**	507	305	0	0	59	47	3	1											SD (3)
**21**	31	25	0	0	4	4	6	3	702	471	3	1							SD (5)

Presented are the baseline values and the values till the completion of the 8^th^ cycle.

^a^MV: missing value

**Fig 5:**
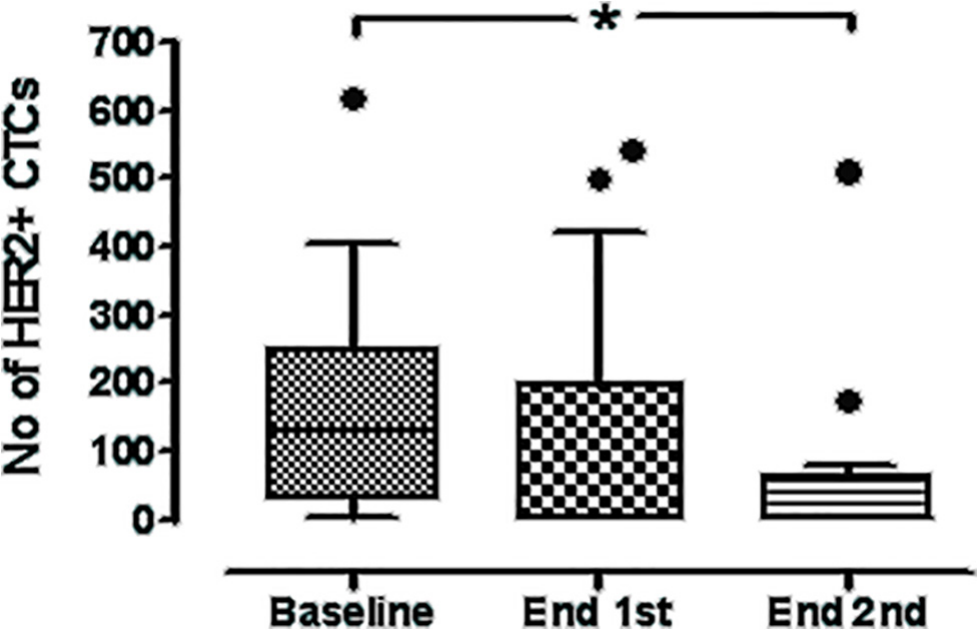
Box-plot of total HER2-positive CTC counts detected in the whole group of patients at baseline and after the completion of the first and second treatment cycles. Shown are the median values ± SD of HER2-positive CTC numbers. HER2-positive CTCs declined from a median of 130 per patient (range 1–617), to a median of 2 (range 0–542) (p = 0.103) and 2 per patient (range 0–513) at the end of the first and second treatment courses, respectively (p = 0.013). Boxes represent the 25^th^ and 75^th^ percentile; line within the box shows the median value (Whiskers: Tukey).

Two patients had HER2-positive primary tumors and both had received prior trastuzumab-based therapy (# 8 and #12, Table 1). In both patients CTC levels were also reduced significantly by the end of the first and second cycle of treatment (Table 2). After the completion of the first and second cycles of treatment, a median of 2 (range 0–542) and 2 (range 0–513) HER2-positive CTCs were identified per patient, respectively (p = 0.103 and p = 0.013, post-first and post-second cycles, respectively, compared to the baseline values) (Fig 5). On the other hand, no difference in the median number of HER2-negative CTCs per patient was evident between baseline and post-first (p = 0.625) or post-second cycle (p = 0.156).

### Effect of lapatinib on CTCs according to outcome

**Fig 6:**
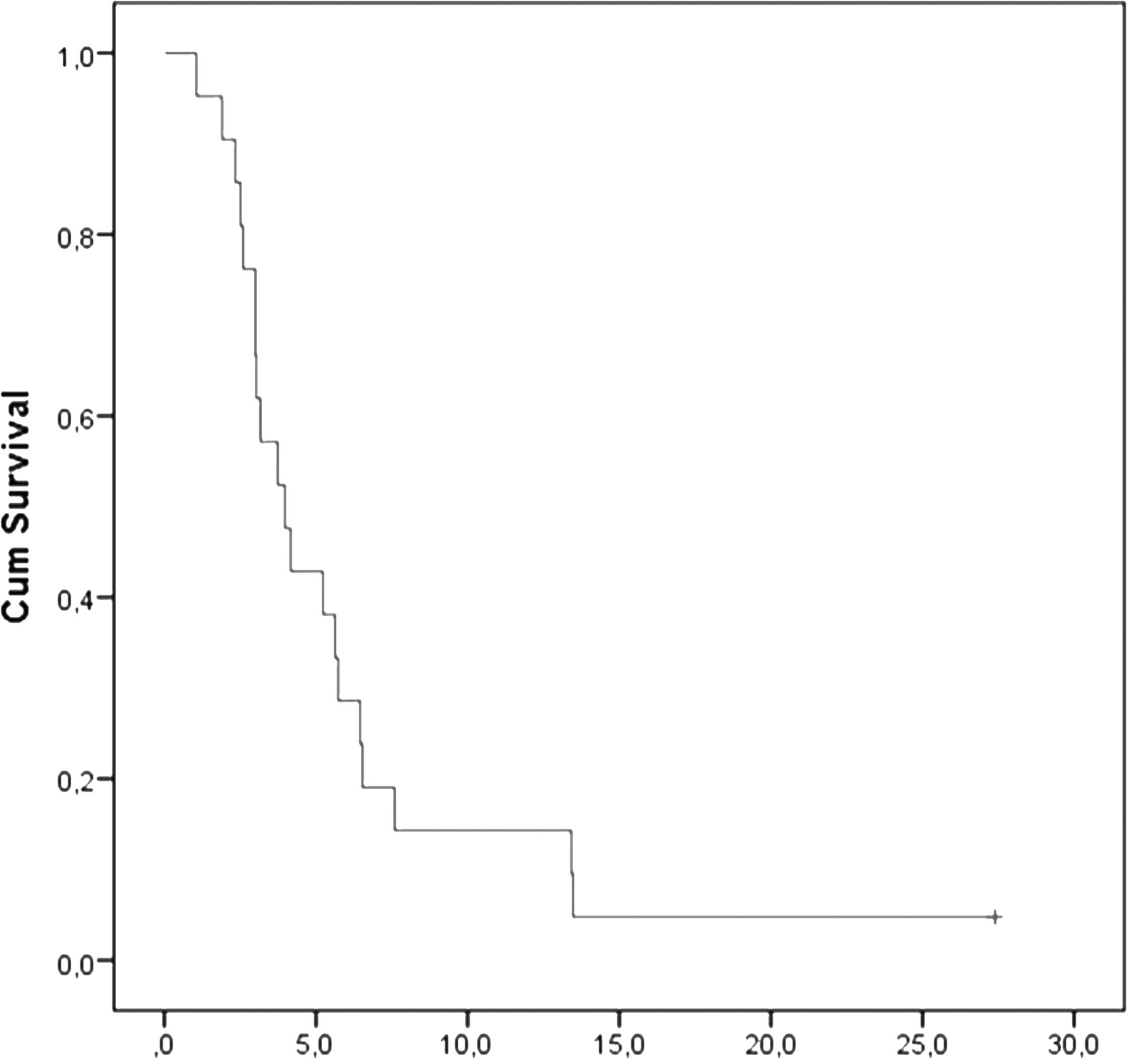
Kaplan-Meier estimate of Progression-Free-Survival (PFS) in patients with metaststic breast cancer receiving lapatinib (n = 21).

A total of 120 treatment cycles were administered (median 4 cycles per patient, range 0.6–27.4). At the time of data cut-off, treatment had been discontinued in 12 (57%) patients due to disease progression, in six (28.6%) due to CTC progression, in one (4.8%) due to both clinical and CTC progression and in one due to toxicity; one patient was still on treatment and one withdrew consent after 3 cycles prior to the documentation of disease or CTC progression. Disease evaluation, revealed stable disease as best response in 11 (52.4%) patients (95% C.I: 31.02%-73.74%) and progressive disease in 10 (47.6%) (Table 2); no objective responses to lapatinib treatment were observed. Median PFS was 4.0 months (range, 1.1–27.4 months, 95% CI: 2.471–5.462) and the 1-year PFS rate was 14.3%. A Kaplan-Meier estimate of PFS is presented as a curve in Fig 6. In seven (33.3%) of 21 patients treatment was administered for a period of 6–27.4 months (Table 2).

**Fig 7:**
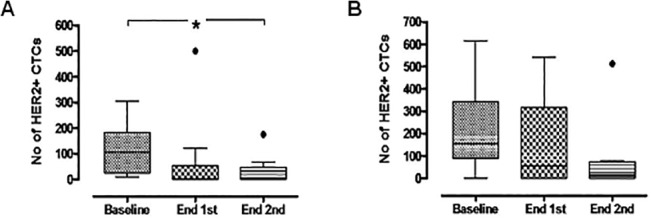
Box-plot of total HER2-positive CTC counts detected in patients with stable disease (A) and progressive disease (B) as best response to treatment at baseline and after the completion of the first and second treatment cycles. Shown are the median values ± SD of HER2-positive CTC numbers. In patients with stable disease CTCs declined from a median of 105 per patient (range 10–305) at baseline to 0 (range 0–500) and 0 (range 0–175), post-first (p = 0.193) and post-second cycles (p = 0.018), respectively. No significant differences in CTC counts were observed in patients with progressive disease. Boxes represent the 25^th^ and 75^th^ percentile; line within the box shows the median value (Whiskers: Tukey).

In patients with clinically stable disease, a median of 105 HER2-positive CTCs per patient (range 10–305) were detected at baseline. After the completion of the first and second cycles of treatment, HER2-positive CTC counts declined to a median of 0 per patient at both time-points (range 0–500 and 0–175, respectively) (post-first, p = 0.193 and post-second, p = 0.018) (Fig 7A). On the contrary, in patients with clinical disease progression, no statistically significant differences were observed between the baseline HER2-positive CTC counts and the counts after the completion of the first (p = 0.275) or second (p = 0.250) cycles of treatment (Fig 7B).

### Evaluation of HER2 and EGFR expression on CTCs at baseline and on progression

HER2 and EGFR expression on CTCs was evaluated at baseline and on progression (either clinical or CTC progression) in 19 out of 21 patients; one patient was still on treatment at the time of data cut-off and the other withdrew consent while clinically stable and in CTC response. The number of HER2-positive CTCs remained decreased at the end-of-treatment sample (median 95 per patient, range 0–513) albeit the difference was not significant compared to the baseline values (p = 0.409). The mean percentage of HER2-negative CTCs increased from 2.4% (range, 0–19.4%) per patient to 30.6% (range, 0–100%) in the end of treatment sample (p = 0.03). In addition, 21.05% of patients harbored HER2-negative CTCs at baseline, compared to 52.94% on progression (p = 0.667), whereas, seven (36.8%) patients had exclusively HER2-negative CTCs. Moreover, 38.04% of the total CTCs detected on progression were HER2-negative (compared to only 1.4% at baseline).

Separate cytospins were evaluated at the same time-points for EGFR expression; all CTC-positive patients had EGFR-positive CTCs on progression compared to 62.5% at baseline (p = 0.054). In addition, both the median number as well as the median percentage of EGFR-positive CTCs per patient increased from 1 and 2% at baseline, to 4 and 87% on the progression sample, respectively, albeit the differences were not statistically significant. Similarly, the ratio of EGFR-positive CTCs/total CTCs detected in all patients increased from 17.1% at baseline to 37.6% on progression.

### Toxicity

Twenty-two patients were evaluable for toxicity. The most common adverse events of any grade were fatigue, diarrhoea and rash reported in 10 (45.4%), 12 (54.5%) and seven (31.8%) patients, respectively (S1 Table). Nausea and vomiting occurred in six (27.3%) patients, whereas paronychia and epistaxis was recorded in four (18.2%) patients each. In general, the toxicity profile of the regimen was easily manageable. One patient presented colitis and gastritis of grade III on day 17 of cycle 1 that persisted despite dose reduction. In this patient, treatment was discontinued before the completion of the first cycle. Another patient withdrew consent after the completion of cycle 3 because of rash, stomatitis and diarrhea all grade II in severity.

## Discussion

CTCs have been shown to persist after previous therapy both in early and metastatic disease and their presence is predictive of subsequent disease recurrence [19,20,24]. These cells are deemed resistant to treatment and are possibly involved in tumor regrowth. Therefore, there is an unmet need to identify effective therapies and new therapeutic targets in patients with “therapy-resistant” CTCs.

A significant discordance between the molecular profile of the primary tumor and corresponding metastases [25,26] as well as between the primary tumor and corresponding CTCs [12–15,27], has questioned the conventional approach of treatment administration according to the characteristics of the primary tumor. In particular, it has been shown that HER2 amplification may be acquired during breast cancer progression [12], whereas in other studies, clinically relevant discrepancies in HER2 expression on CTCs and in corresponding primary tumors were reported [13,15,27]. On the other hand, CTCs are increasingly considered as a “liquid biopsy” of the tumor at the time of blood sampling whereas HER2-positive CTCs have been associated with poor clinical outcome in early breast cancer [17,28,29]. We have previously demonstrated the effectiveness of trastuzumab in eliminating CK-19 mRNA-positive CTCs in breast cancer patients after prior chemotherapy exposure [30]. In the current study we further demonstrate that lapatinib is effective in decreasing HER2-positive CTC counts in patients with MBC irrespectively of the HER2 status of the primary tumor.

The decline in the levels of HER2-positive CTCs was an early effect of lapatinib treatment since by the end of the first treatment cycle a reduction was observed in 14 (66.6%) patients and the median time to nadir CTC levels was 1 month (range, 1–5 months). Moreover, lapatinib effectively decreased CTC counts in 2 patients with HER2-positive tumors despite prior administration of trastuzumab-based therapy which could influence the effect of lapatinib. This finding suggests that lapatinib can effectively target CTCs presenting resistance to trastuzumab. Similarly, in preclinical studies, lapatinib was effective against breast cancer with resistance to trastuzumab [31] whereas, lapatinib added to capecitabine provided superior efficacy compar1ed to capecitabine alone in women with HER2-positive, MBC progressing after prior trastuzumab-based therapy [32].

It should be noted here that any alteration in CTC counts compared to the baseline values was considered as a change and that this trial was not designed to determine which level of CTC change could be clinically relevant. Moreover, due to the small number of patients included in this trial, the above results need to be confirmed in future studies.

The number of HER2-positive CTCs detected in the whole group of patients was also reduced significantly after the end of the second cycle of treatment. This effect was specific for the HER2-positive CTCs, since HER2-negative CTC counts were not significantly altered. When CTC kinetics was evaluated separately in patients with disease stabilization or disease progression, it was shown that HER2-positive CTCs declined significantly only in patients with stable disease. This observation indicates that disease stabilization could be related to the depletion of the HER2-positive CTC population during treatment with lapatinib.

The monitoring of the molecular changes on CTCs during treatment with targeted agents provides the unique opportunity to investigate the mechanisms that contribute to drug resistance [33]. In the current study, the pool of HER2-positive CTCs was numerically lower in the samples obtained on progression compared to the pre-treatment sample, although this difference was not statistically significant; on the other hand, HER2-negative CTCs counts increased during progression. These observations suggest the emergence of a population of lapatinib-resistant HER2-positive and HER2-negative CTCs potentially associated with disease progression.

When EGFR expression was evaluated at the same time-points, it was shown that more patients harbored EGFR-positive CTCs on disease progression compared to the pre-treatment sample and this difference showed a trend for statistical significance. In addition, the percentage of EGFR-positive CTCs per patient, as well as the ratio of EGFR-positive CTCs among the total CTCs detected, was increased at the end-of-treatment sample. It has been shown that lapatinib lacks efficacy in HER2-negative, EGFR-overexpressing inflammatory breast cancer [34]. In our study, the pool of HER2-negative CTCs was increased in the blood samples obtained on progression. Thus, one could postulate that EGFR-positive/HER2-negative CTCs may contribute to lapatinib resistance.

No objective responses were observed in this cohort of patients. However, disease and/or CTC-stabilization of 6–27 months duration were evident in seven out of 21 patients. Similarly, lapatinib failed to induce clinical responses in patients with HER2-negative MBC and HER2-positive [35] or EGFR-positive [36] CTCs. However, in the aforementioned study, one of seven patients with HER2-positive CTCs presented disease stabilization lasting for 8.5 months [35]. In these trials lapatinib was administered in MBC patients progressing after prior treatment. In addition, CTC characterization was performed by immunofluorescence staining for HER2 or EGFR by using the fourth spare filter of the CellSearch System. In this trial we demonstrate the feasibility of targeting CTCs resistant to previous therapy in non-progressing patients using a CTC-directed approach as maintenance therapy. In addition, HER2 characterization was performed by immunofluorescent staining of CTCs isolated by density gradient centrifugation of peripheral blood and not selected based on EpCam-positivity as is the case of the CellSearch system.

A possible limitation of our study is that HER2-positivity was not scored quantitatively; HER2 expression was subjectively characterized as positive or negative according to the expression levels observed in the negative control. Moreover, the cut-off for patient eligibility was arbitrarily set as the detection of at least one HER2-positive CTC. Finally, one could argue that the observed effect of lapatinib on CTCs, does not necessarily imply that the drug is also clinically effective in this setting; the differential kinetics of CTCs in progressing patients compared to those with disease stabilization could simply reflect the natural course of the disease and should not necessarily be assigned to the effect of lapatinib. Nevertheless, it should be also noted that CTCs have been previously demonstrated to be a surrogate marker of treatment efficacy [37].

The failure of lapatinib to induce clinical responses in the present as well in other studies [35,36] designed to target a marker present on CTCs rather than the primary tumor, should not be considered as a failure of this strategy. We have previously shown that the administration of 6 cycles of trastuzumab in patients with HER2-negative early breast cancer harbouring CK-19mRNA-positive CTCs both before and after the completion of adjuvant chemotherapy, was associated with a significantly lower incidence of clinical relapses and a longer disease-free survival compared to patients who received the standard of care [38]. Larger studies such as the TREAT CTC trial (NCT01548677) in early breast cancer and the DETECT III trial (NCT01619111) in metastatic disease, will further investigate whether treatment individualization according to the molecular characteristics of CTCs could be a practice changing approach in breast cancer.

In conclusion, the results of this study demonstrate that lapatinib is effective in decreasing HER2-positive CTC counts in patients with MBC, and that this effect is correlated with disease stabilization. Furthermore they show that HER2-negative CTC counts increase on disease progression. The above observations imply that CTC isolation and molecular characterization could be a novel tool for the real time phenotyping of tumor cells during treatment with targeted agents. Moreover, our results suggest that tailoring therapy according to targets present on CTCs could be clinically relevant in patients with breast cancer. To this end, standardized assays and validated cut-offs should be established in order to use these targets for the individualization of treatment.

## Supporting Information

S1 CONSORT Checklist CONSORT checklist. (DOC)

S1 Protocol Trial Protocol. (DOC)

S1 Table All grades toxicity observed in all patients and all cycles (n = 22). (DOC)
