# Supplementary material for: Efficacy of Lapatinib in Therapy-Resistant HER2-Positive Circulating Tumor Cells in Metastatic Breast Cancer
Source: PLoS One. 2015 Jun 17;10(6):e0123683. doi: 10.1371/journal.pone.0123683 (PMC4471111; doi:10.1371/journal.pone.0123683)
Supplement: S1 Protocol — (DOC) [file pone.0123683.s002.doc]

**A pilot feasibility study to evaluate the efficacy of lapatinib in eliminating cytokeratin-positive tumour cells circulating in the blood of women with breast cancer**

Study Code : CT/07.15

Version : 1.0, Final

Sponsor : Prof. Vassilis GEORGOULIAS, MD, PhD

School of Medicine, University of Crete

EudraCT no : 2007-005400-40

Principal Investigator : Sofia Agelaki MD, PhD

Dpt of Medical Oncology

University Hospital of Heraklion

Study Chairman : Vassilis Georgoulias MD, PhD

Professor of Medical Oncology

Dpt of Medical Oncology

University Hospital of Heraklion

| **TABLE OF CONTENTS** | **PAGE** |
| --- | --- |

[Protocol synopsis 5](#__RefHeading___Toc56920072)

[1. Introduction 7](#__RefHeading___Toc56920074)

[1.1 Breast Cancer 7](#__RefHeading___Toc56920075)

[1.2 Disseminated tumor cells in patients with breast cancer. Clinical significance](#__RefHeading___Toc56920076)  7

1.3 Lapatinib 9

1.3.1 Background 9

1.3.2 Preclinical Data 10

1.3.3 Clinical Data 11

1.3.3.1 Efficacy of lapatinib administered as monotherapy in patients with breast cancer.11

1.3.3.2 Safety of lapatinib 14

1.3.3.2.1 Serious adverse events 16

1.3.3.3 Biomarkers 22

1.3.4 Clinical pharmacokinetics 22

1.4 Rationale for this study 22

[2. STUDY OBJECTIVES 24](#__RefHeading___Toc56920077)

[2.1 Primary objective 24](#__RefHeading___Toc56920078)

[2.2 Secondary objectives 24](#__RefHeading___Toc56920079)

[2.3. Exploratory Objective 25](#__RefHeading___Toc56920080)

[3. Study plan and procedures 25](#__RefHeading___Toc56920081)

[3.1 Overall study design and flow chart 25](#__RefHeading___Toc56920082)

[3.2 Rationale for study design, doses](#__RefHeading___Toc56920083) 29

[3.3 Selection of study population 29](#__RefHeading___Toc56920084)

[3.3.1](#__RefHeading___Toc56920085) [Inclusion criteria 29](#__RefHeading___Toc56920086)

[3.3.2 Exclusion criteria 31](#__RefHeading___Toc56920087)

[3.3.3 Discontinuation of subjects from treatment or assessment 31](#__RefHeading___Toc56920089)

[3.3.3.1 Criteria for Discontinuation 31](#__RefHeading___Toc56920090)

[3.3.3.2 Procedures for discontinuation 34](#__RefHeading___Toc56920091)

[3.4 Treatments 34](#__RefHeading___Toc56920092)

[3.4.1 Investigational products 34](#__RefHeading___Toc56920093)

[3.4.1.1](#__RefHeading___Toc56920094) [Doses and treatment regimens 34](#__RefHeading___Toc56920095)

[3.4.1.2](#__RefHeading___Toc56920096) [Storage 35](#__RefHeading___Toc56920097)

[3.4.1.3 Accountability 35](#__RefHeading___Toc56920098)

[3.4.2](#__RefHeading___Toc56920099) [Pre-study, concomitant and post-study treatment(s) 36](#__RefHeading___Toc56920101)

[3.4.2.1 Treatment for cancer 36](#__RefHeading___Toc56920102)

[3.4.2.2 Lapatinib Prohibited Medication List 36](#__RefHeading___Toc56920103)

[3.5 Management of Toxicity 38](#__RefHeading___Toc56920104)

[3.5.1 Criteria for evaluation cardiac and respiratory events 38](#__RefHeading___Toc56920105)

3.5.1.1 Symptomatic events 38

3.5.1.2 Symptomatic cardiac events 38

[3.5.2 Rash](#__RefHeading___Toc56920106) 39

[3.5.3](#__RefHeading___Toc56920107) [Diarrhea 3](#__RefHeading___Toc56920109)9

[3.5.4 Nausea 4](#__RefHeading___Toc56920110)0

[3.6 Dose delays/dose modifications 4](#__RefHeading___Toc56920111)1

[3.6.1 Dose delays and dose reductions](#__RefHeading___Toc56920112) 41

[4. REGULATORY AND REPORTING REQUIREMENTS](#__RefHeading___Toc56920115) 45

[4.1 Assessment of causality](#__RefHeading___Toc56920116) 45

[4.2](#__RefHeading___Toc56920117) Following up of adverse events 46

4.3 [Definitions of serious adverse events](#__RefHeading___Toc56920127) 46

[4.3.1 Life treatening definition](#__RefHeading___Toc56920128) 47

[4.3.2 Disability/incapacitating definition](#__RefHeading___Toc56920129) 47

[4.3.3 Hospitalization definition 4](#__RefHeading___Toc56920130)7

4.3.4 Additional SAE definitions 47

4.4 Reporting serious adverse events 48

4.4.1 Lack of efficacy 48

4.5 Pregnancy 50

References 51

Appendix A: World Medical Association Declaration of Helsinki 55

Appendix B: Informed Consent Form sample 60

| **LIST OF IN-TEXT TABLES** | **PAGE** |
| --- | --- |

[Table 1 Overall response rate and clinical benefit rate. Study EGF20002…………](#__RefHeading___Toc33496426)…….10

[Table 2 Overall response rate and clinical benefit rate](#__RefHeading___Toc33496427). Study EGF20008. 11

Table 3 Summary of AEs related to study medication reported in 10% or more of subjects in either treatment group in the lapatinib monotherapy-based clinical trials EGF20002, EGF20008, EGF20003, EGF20004 (Safety Population) ………......13

## Table 4. Study design and flow chart………………………………………………………21

Table 5 Study plan…………………………………………………………………..........22

Table 6 Lapatinib starting dose and dose reduction schedule…………………………….32

Table 7 Dose reduction criteria and guideline for management of lapatinib associated toxicity…………………………………………………………………………...32

Protocol synopsis

**A pilot feasibility study to evaluate the efficacy of lapatinib in eliminating cytokeratin-positive tumour cells circulating in the blood of women with breast cancer**

Principal Investigator Sofia Agelaki MD, PhD

Dep of Medical Oncology

University Hospital of Heraklion

Study Chairman: Vassilis Georgoulias MD, PhD

Professor of Medical Oncology

Dep of Medical Oncology

University Hospital of Heraklion

Study centre(s) and number of subjects planned

This study will be conducted in the Department of Medical Oncology at the University General Hospital of Heraklion, in Greece. Twenty women with metastatic breast cancer are planned to participate in this study.

Objectives

To evaluate the efficacy of lapatinib in eradicating chemo- resistant tumour cells circulating in the blood of patients with breast cancer. These cells will be detected using immunofluorescence microscopy on Peripheral Blood Mononuclear Cell (PBMC) cytospins.

Study design

Open label, single-arm exploratory phase II clinical trial

Target subject population

Patients with metastatic breast cancer who have persistent detection of tumour cells in the peripheral blood (defined as ≥1 CTC per 10^6^ PBMCs detected by immunofluorescence microscopy) despite at least one line of chemotherapy will receive lapatinib for a minimum of one month unless disease progression (defined by RECIST) occurs at an earlier time point. Prior hormonal therapy administered for metastatic disease is allowed.

Investigational product, dosage and mode of administration

Lapatinib 1500 mg/day orally

Duration of treatment

All patients will receive lapatinib for a minimum of one month unless clinical disease progression (defined by RECIST criteria) occurs at an earlier time point. At the end of the 1-month period patients will be assessed for the presence of CTCs in peripheral blood. Those with decreased or stable CTC counts will continue treatment and will be assessed every month. Patients with increased CTC counts will be withdrawn from the study if further increase is documented at re-assessment after the second treatment course. In subsequent cycles, treatment will be also discontinued in case of two consecutive increases in CTC values. Moreover, treatment will be discontinued in case of disease progression, occurrence of unacceptable toxicity or consent withdrawal, irrespectively of CTC counts.

Outcome variables

**Efficacy**

- **Primary outcome variable**:
- Effect of lapatinib on HER2-positive CTC counts.
- **Secondary outcome variables:**
- Correlation of the kinetics of CTCs with response to treatment
- Safety of lapatinib treatment in this patient population

**Exploratory**

- Detection of cytokeratin-positive cells
- Detection of cytokeratin-positive/EGFR-positive CTCs

#

# 1. Introduction

**1.1 Breast cancer**

Breast cancer is the most common cancer among women and is the second leading cause of female death (American Cancer Society, 1994). Although a small percentage of patients with metastatic breast cancer achieve long-term disease-free survival, the disease at this stage is not curable and therapy is only palliative.

## Over the last two decades, chemotherapy and endocrine therapy have become the most important therapies in the treatment of patients with early or metastatic breast cancer. Adjuvant chemotherapy and/or endocrine therapy represent the standard of care for women with early breast cancer expressing hormone receptors. In the metastatic setting endocrine treatments are the therapy of choice for asymptomatic, non-life-threatening disease in patients with oestrogen receptor and/or progesterone receptor (ER/PgR)-positive tumours; chemotherapy has to be used if metastases become symptomatic or if specific organ function is impaired. Surgery or radiotherapy is confined to specific indications and is mainly used in combination with systemic treatment.

**1.2 Disseminated tumor cells in patients with breast cancer. Clinical significance**

Breast cancer is considered a systemic disease since early tumour cell dissemination may occur even in patients with small tumours; several investigators using immunocytochemistry or real time PCR (RT-PCR) have shown that cytokeratin-positive epithelial cells could be identified in the bone marrow aspirates or the peripheral blood of otherwise metastases-free patients with stage I and II breast cancer (1,2). The clinical importance of the immunocytochemical detection of occult tumour cells in the bone marrow has been confirmed in many prospective studies to represent an independent predictive and prognostic factor for distant relapse and reduced survival (3-6).

A molecular methodology to detect occult tumour cells is based on the amplification of cytokeratin 19 by RT-PCR (CK-19) mRNA; this assay is capable of detecting one tumour cell among one million normal cells and is at least 10 times more sensitive than immunocytochemistry (7). Indeed, occult tumour cells have already been identified by nested RT-PCR in the peripheral blood of breast cancer patients by many investigators (8-11).

Our group has recently evaluated the presence and the prognostic significance of circulating tumour cells (CTCs) expressing the CK-19 mRNA by nested RT-PCR in the peripheral blood of 148 patients with stage I and II breast cancer before the initiation and after the completion of adjuvant chemotherapy (11, 12). CK-19 mRNA-positive cells could be detected in almost 30% of patients before adjuvant chemotherapy, and multivariate analysis demonstrated that the presence of these cells was an independent prognostic factor for decreased DFI (p=0.001) and overall survival (p=0.01). Moreover, adjuvant chemotherapy failed to eliminate the circulating CK-19 mRNA-positive cells in almost half of the patients while an additional 20% of patients developed CK-19 mRNA-positive cells in the blood during the treatment. The risk of relapse was 3.8 (95% C.I. 1.06-13.56) times higher and the DFI was significantly reduced (p=0.03) if CK-19 mRNA-positive cells were detectable after the completion of adjuvant chemotherapy in patients with <3 involved axillary lymph nodes. In a recently completed study our group showed that the prognostic significance of the detection of circulating CK-19 mRNA-positive cells is also applied in patients with disease stage I (13). Similarly to our findings, Braun et al (14), using an immunocytochemical technique, studied 23 patients with inflammatory and 36 with operable breast cancer who had more than 4 axillary lymph nodes involved, for the presence of occult bone marrow breast cancer cells before and after the completion of adjuvant chemotherapy; they found that only 48.3% of the patients who had CK-positive cells in the bone marrow before treatment became CK-negative post-treatment; multivariate analysis showed that the detection of CK-positive cells post-chemotherapy represents an independent poor prognostic factor for reduced overall survival.

CTCs have been also identified in 40-60% of patients with metastatic breast cancer (15-16). Using a newly developed CellSearch system (Veridex) that was designed to detect tumor cells in peripheral blood, Cristofanilli et al (16) showed that the level of CTCs before a new treatment is initiated as well as the level measured three to four weeks after treatment initiation, are useful predictors of progression-free and overall survival. Specifically, patients with levels of CTCs equal to or higher than 5 per 7.5 ml of whole blood as compared with the group with fewer than 5 CTCs per 7.5 ml, had a worse prognosis. Moreover, the outcome of patients with ≥5 CTCs at baseline but <5 at the first follow-up visit was comparable to that of patients with <5 CTCs both at the the first follow-visit and at baseline.

The above findings imply the significance of CTCs as a prognostic tool in cancer treatment. However, since the presence of CTCs is the necessary (although not sufficient step) in the metastatic process, these cells represent the subset of tumor cells that lead to the development of metastasis. Metastatic cells can have a very different molecular profile from the tumor of origin, therefore CTCs might theoretically be used for the identification of the molecular signature of the specific tumor over time. Thus, chemotherapeutic drugs and molecularly targeting agents might be more efficiently tested in CTCs rather than the primary tumor.

##

## 1.3 Lapatinib

1.3.1 Background

Lapatinib acts as a dual inhibitor of both EGFR and ErbB2 tyrosine kinase activity. As a member of the 4-anilinoquinazoline class of kinase inhibitors, lapatinib is thought to react with the ATP binding site of EGFR/ErbB2, resulting in inhibition of autophosphorylation and subsequent proliferative signalling (17). The ErbB family consists of four closely related growth factor receptor tyrosine kinases. The family is comprised of ErbB1 (EGFR/HER), ErbB2 (HER2/neu), ErbB3 (HER3), and ErbB4 (HER4). All members of the ErbB family share a common extracellular ligand-binding domain, a single membrane-spanning region and a cytoplasmic tyrosine kinase domain (reviewed in 18, 19). A ligand for ErbB2 has not been identified, while ErbB3 lacks tyrosine kinase activity. Ligand binding to EGFR, ErbB3 or ErbB4 induces these inactive monomers to undergo an array of homo- or heterodimerization with other members of the ErbB family. ErbB2 is the preferred heterodimeric partner for all ErbB receptors, resulting in a complex that is endocytosed at one half to one third the rate of other EGFR dimers (20, 21). ErbB dimerization leads to receptor autophosphorylation and subsequent activation of the tyrosine kinase domain. The signalling characteristics of the ErbB family are thought to be strongly interdependent.

EGFR expression by tumor cells has been linked with aggressive tumor growth, disease progression, poor survival, and poor response to therapy. Over-expression of EGFR has been reported in a number of epithelium-derived carcinomas including head and neck, colorectal, lung, esophageal, gastric, and breast carcinoma. In a similar manner, ErbB2 has been reported to be over-expressed in 15-30% of invasive ductal breast cancer and has been associated with increased proliferation, poor clinical outcome, and altered responsiveness to various adjuvant therapies (22, 23). Activation of either EGFR or ErbB2 initiates a series of signalling cascades that includes mitogen-activated protein kinase (MAPK), phosphoinositide 3-kinase (PI3K), Akt, and p70S6K.

**1.3.2 Preclinical Data**

The ability of lapatinib to inhibit the growth of EGFR-over-expressing cell lines (IC_50_ <0.16 µM) was observed to be equal to that of other EGFR inhibitors being tested in clinical trials (e.g. gefitinib/Iressa^TM^ or erlotinib/Tarceva^TM^). With the exception of ErbB4, lapatinib was >300-fold more selective towards ErbB2 and EGFR kinase inhibition than to other kinases tested. Lapatinib demonstrated potent growth inhibition of human breast ductal (BT474) and head and neck (HN5) tumor xenografts in mice. A dose response inhibition was observed in both models receiving lapatinib (30 or 100 mg/kg twice daily orally for 21 days). Complete inhibition of tumor growth was seen in mice receiving 100 mg/kg (24).

Lapatinib has been shown to inhibit Erk1/2 and Akt phosphorylation (pErk and pAkt) in both EGFR and erbB2-expressing cell lines (BT474 and HN5). The ability of lapatinib to inhibit pAkt was associated with a 23-fold increase in the percentage of cells undergoing apoptosis compared to control cells. Similarly, lapatinib treatment of BT474 and HN5 xenografts in mice also resulted in inhibition of Erk1/2 and Akt phosphorylation. These results suggested that lapatinib treatment of EGFR/erbB2 expressing tumors could lead to inhibition of downstream signalling events (25).

A study of human breast cancer cell lines that over-express EGFR or erbB2 (SUM102, SUM149, SUM185, and SUM225) reported that treatment with lapatinib resulted in inhibition of cell proliferation that was associated with inhibition of Erk phosphorylation. Inhibition of Erk phosphorylation also correlated with radiosensitization of cell lines pretreated with lapatinib (26).

**1.3.3 Clinical Data**

**1.3.3.1 Efficacy of lapatinib administered as monotherapy in patients with breast cancer**

**Completed monotherapy studies in the treatment of breast cancer**

Completed Monotherapy Study EGF20002 (27) was conducted in female subjects with advanced (Stage IIIb) or metastatic (Stage IV) breast cancer who had progressed while receiving trastuzumab-containing regimens (monotherapy or combined therapy). Table 1 presents a summary of the efficacy results for the primary endpoints of overall response rate and clinical benefit rate using RECIST criteria. The independent review was independent of subject management and blind to the response evaluations of the investigator review.

**Table 1. Overall Response Rate and Clinical Benefit Rate, Study EGF20002**

|  | **Lapatinib 1250 mg/1500 mg QD**  **N=78** | |
| --- | --- | --- |
| **Response, n (%)** | **Independent Review** | **Investigator Review** |
| **Best Response** |  |  |
| Complete Response (CR) | 0 | 1 (1) |
| Partial Response (PR) | 4 (5) | 5 (6) |
| Stable Disease | 31 (40) | 29 (37) |
| Progressive Disease | 26 (33) | 40 (51) |
| Unknown | 17 (22) | 3 (4) |
| Response Rate (CR or PR)^1^ | 5,1 % | 7,7% |
| Clinical Benefit Rate^2^ | 9,0% | 14,1% |

1. Tumor response rates in subjects with tumor tissues confirmed as FISH-positive and/or IHC 3+ for ErbB2 as

evaluated by the independent review panel and by the investigator were 3.9% and 7.8%, respectively.

2. CR or PR or Stable Disease ≥24 weeks

A total of nine subjects had a positive tumor response (complete response [CR] or PR) based on either independent or investigator review. For all responders, the first tumor responses were documented between Week 8 and Week 16. The duration of response ranged from 50 to 113 days based on the independent review and from 60 to 292 days based on the investigator review. Median time to tumor progression was 15.3 weeks based on independent review and 9 weeks based on the investigator review. Median progression-free survival interval was 15.3 weeks based on independent review and 9 weeks based on the investigator review. Based on independent review, the Kaplan-Meier estimated probability of being progression-free at 4 months and at 6 months was 41% and 21%, respectively. Statistical analyses for 4-month and 6-month progression-free survival were not performed based on the investigator review. Median survival duration was 78.6 weeks. The probabilities of surviving at 4 months and at 6 months were 89% and 85%, respectively.

Completed Monotherapy Study EGF20008 (27) was conducted in female subjects with advanced (Stage IIIb) or metastatic (Stage IV) breast cancer refractory to treatment with anthracycline-, taxane- and capecitabine-containing regimens. Subjects enrolled in Cohort A had ErbB2 overexpressing tumors and were refractory to treatment with trastuzumab-containing regimens. Subjects enrolled in Cohort B had ErbB2 non-overexpressing tumors and had no prior treatment with trastuzumab. Table 2 presents a summary of the efficacy results for the primary/secondary endpoints of overall response rate and clinical benefit rate using RECIST criteria. The independent review was independent of subject management and blind to the response evaluations of the investigator review.

A total of seven subjects in Cohort A had a positive tumor response (CR or PR) based on either independent panel or investigator review. A statistical analysis of time to tumor response and duration of tumor response was not conducted due to an insufficient number of responders. Of the seven subjects who had a response, first response was documented at Week 4 (Day 29) for one subject, Week 8 for five subjects (Days 50-57), and Week 16 (Day 113) for one subject. The duration of response ranged from 55 to >250 days (8 to >36 weeks).

**Table 2. Overall Response Rate and Clinical Benefit Rate, Study** **EGF20008**

|  | **Lapatinib 1500mg QD** | | | |
| --- | --- | --- | --- | --- |
|  | **Cohort A**  **N=140** | | **Cohort B**  **N=89** | |
| **Overall Response Rate,**  **n (%)** | **Independent Review** | **Investigator Review** | **Independent Review** | **Investigator Review** |
| **Best Response** |  |  |  |  |
| Complete Response (CR) | 0 | 3(2) | 0 | 0 |
| Partial Response (PR) | 2 (1) | 3 (2) | 0 | 0 |
| Stable Disease | 46 (33) | 38 (27) | 10 (11) | 10 (11) |
| Progressive Disease | 64 (46) | 85 (61) | 49 (55) | 76 (85) |
| Unknown | 28 (20) | 11 (8) | 30 (34) | 3 (3) |
| Tumor Response Rate | 1,4% | 4,3% | 0% | 0% |
| Clinical Benefit Rate^1^ | 5,7% | 5,7% | 0 | 0 |
| Clinical Benefit Rate^2^ | 15,7% | 10,7% | 1,1% | 1,1% |

1. Defined as CR or PR or Stable Disease ≥24 weeks

2. Ad-hoc analysis defined as CR or PR or Stable Disease ≥16 weeks

The median time to tumor progression was 9.1 weeks in Cohort A and 7.6 weeks in Cohort B based on independent review. The median time to tumor progression was 8.1 weeks in Cohort A and 7.0 weeks in Cohort B based on investigator review.

The median progression-free survival interval was 9.1 weeks in Cohort A and 7.6 weeks in Cohort B based on independent review. The Kaplan-Meier estimated probability of being progression-free at 4 months and at 6 months was 34% and 18%, respectively, in Cohort A, and 7% and 0%, respectively, in Cohort B.

The median survival time was 29.4 weeks for subjects in Cohort A and 18.6 weeks for subjects in Cohort B.

Completed Monotherapy Study EGF20009 (28) was a randomized, open-label, parallel-group, multicenter Phase II study of lapatinib monotherapy in subjects with advanced or metastatic breast cancer whose tumors overexpress ErbB2 and who have not had prior systemic therapy for advanced or metastatic disease or local recurrence, except hormonal therapy. Subjects were randomized into one of two dose groups: lapatinib 500 mg BID or lapatinib 1500 mg QD. Subjects were treated until disease progression or unacceptable toxicity. 138 subjects were evaluable for the primary endpoint of tumor response (69 subjects in the 500 mg BID group and 69 subjects in the 1500 mg QD group). Disease assessments, based on images and medical photographs, were completed by the investigators and an independent review board. There were no complete responses, as assessed by the independent review board, in either treatment group, but 2 and 1 CRs were observed by the investigators’ review in the 500 mg BID and 1500 mg QD groups respectively. There were 33 (24%) confirmed partial responses by RECIST criteria, as assessed by the independent review board. The clinical benefit rate (CR + PR + SD > 24 weeks) was 31,2%. Median progression free survival was 15,7 weeks on 500 mg BID and 17 weeks on 1500 mg QD. There was no difference in the efficacy parameters between arms.

**1.3.3.2. Safety of lapatinib**

##### For further safety information see the current Investigator Brochure.

A phase I study of lapatinib at doses of 175 to 1800 mg/day in heavily pre-treated patients (n=39) with solid tumors reported that the drug was well-tolerated and showed preliminary evidence of antitumor activity. In this study, 3 patients in each of the 175 and 375 mg/day cohorts; 4 in the 675, 900, and 1600 mg/day cohorts; 6 in the 1200 mg/day and 9 in the 1800 mg/d were enrolled. Six additional patients were administered 900 mg bid to compare safety of bid versus qday schedules. Grade 1-2 rash, diarrhea, nausea, vomiting, constipation, fatigue, and anorexia were the most frequent adverse events in all qday dose cohorts. Grade 3 toxicity was not observed in any of the qday dose cohorts. Grade 3 diarrhea was observed in 2 of 6 patients at 900 mg bid, requiring dose reductions. Eight patients (non-small cell lung cancer (NSCLC), carcinoma of unknown primary site, head and neck, colon, breast carcinomas) remained on study with stable disease (SD) of 4+ months duration. Two NSCLC patients resistant to gefitinib (ZD1839, Iressa™) had minor responses (MRs) (3+ months, 12 months). Patients continuing on therapy for more than 4 months had received lapatinib at doses > 1200 mg/day. Lapatinib was well tolerated when administered on a qday schedule, with preliminary evidence of anti-tumor activity in this heavily pre-treated population (29).

Four phase II studies evaluated the open-label use of lapatinib 1250 mg QD or 1500 mg QD as a single-agent in the second-line treatment of advanced or metastatic cancer. The safety information provided is based on the Safety population, consisting of all subjects who received at least 1 dose of study drug.

Two of the 4 completed studies evaluated the safety and efficacy of lapatinib 1250 mg QD to 1500 mg QD in the treatment of breast cancer [n=78], [n=229] (27). In EGF20002 (27), 34 subjects began treatment at 1250 mg of lapatinib QD. A dose increase to 1500 mg QD was implemented based on findings from a Phase I study, and 7 of those remaining in the study had their dose increased to 1500 mg lapatinib QD. However, for reporting purposes, these subjects are assigned to their original 1250 mg QD dosage. Forty-four subjects began treatment with lapatinib at 1500 mg QD in EGF20002 (27). All 229 subjects enrolled in EGF20008 (27) received 1500 mg QD. Completed study EGF20003 (27) (n=59) evaluated the safety and efficacy of lapatinib 1250 mg QD in the treatment of bladder cancer, and completed study EGF20004 (27) (n=86) evaluated the safety and efficacy of lapatinib 1250 mg QD in the treatment of colorectal cancer.

Across the 4 studies, 452 subjects received open-label lapatinib; 179 (40%) subjects received lapatinib 1250 mg QD and 273 (60%) subjects received lapatinib 1500 mg QD. Based on exposure data available as of the analysis cut-off date (n=179 for the 1250 QD group, n=262 for the 1500 QD group), the median duration of treatment was 8 weeks for both dosage groups. Almost all subjects (97%) received treatment ≥80% of days during the treatment period.

All subjects receiving 1500 mg QD were women; the 2 studies evaluating this dose were breast cancer studies. Approximately half of the 1250 mg QD population was women (49%). The majority of all subjects were white (86%). Black subjects represented 8% of all subjects, and Asian subjects represented 3% of all subjects. The median age was 61.0 years for subjects receiving 1250 mg QD and 53.0 for subjects receiving 1500 mg QD.

The overall incidence of AEs (regardless of relationship to study medication) was similar between the dosage groups; 95% and 97% of subjects in the 1250 mg QD and 1500 mg QD dosage groups reported at least one AE. The most common AEs (≥25% of any group) regardless of relationship to drug were diarrhea (1250 mg QD: 44%, 1500 mg QD: 60%), rash (1250 mg QD: 39%, 1500 mg QD: 36%), nausea (1250 mg QD: 23%, 1500 mg QD: 39%), and fatigue (1250 mg QD: 24%, 1500 mg QD: 31%). The majority of these AEs were Grade 1 or 2 toxicity. These events are included in the core safety information for lapatinib and are consistent with the safety profile of lapatinib as reported for the program as a whole.

The overall incidence of AEs considered by the investigator to be related to study medication was slightly higher in the 1500 mg QD group (82%) compared with the 1250 mg QD group (73%). The most common drug-related AEs (≥10% of any group) were diarrhea, nausea, vomiting, rash (skin disorder), fatigue, and anorexia (Table 3).

**Table 3. Summary of AEs related to study medication reported in 10% or more of subjects in either treatment group in the lapatinib monotherapy-based clinical trials EGF20002, EGF20008, EGF20003, EGF20004 (Safety Population)**

| **Preferred Term** | **Lapatinib 1250 mg QD**  **N=179**  **n (%)** | **Lapatinib 1500 mg QD**  **N=273**  **n (%)** |
| --- | --- | --- |
| **Any Related AE** | 130 (73) | 223 (82) |
| Diarrhea | 62 (35) | 147 (54) |
| Rash | 61 (34) | 89 (33) |
| Nausea | 27 (15) | 74 (27) |
| Vomiting | 19 (11) | 39 (14) |
| Fatigue | 18 (10) | 42 (15) |
| Anorexia | 14 (8) | 27 (10) |

**1.3.3.2.1 Serious Adverse Events**

Preliminary SAE data are available for the lapatinib program (all phases) up to a cut off date of 03 April 2006. At the time of the SAE cut off, 4368 subjects were enrolled in Phase I, II and III studies, of which approximately 3147 subjects have received lapatinib.

A total of 1729 SAEs were reported from 917 individual subjects. The most frequently reported SAE was diarrhea, with a total of 121 reports, 96 of which were assessed as related to investigational product. Vomiting, dyspnea, pyrexia and dehydration were also amongst the most frequently reported SAEs regardless of relatedness. The ten most frequently reported SAEs are listed below:

| **MeDRA PT** | **Drug Related** | **Total (All Causalities)** |
| --- | --- | --- |
| Diarrhea* | 96 | 121 |
| Vomiting* | 36 | 77 |
| Dyspnea | 8 | 56 |
| Pyrexia | 11 | 54 |
| Dehydration* | 21 | 49 |
| Neutropenia^1^ | 39 | 48 |
| Nausea* | 23 | 47 |
| Ejection fraction decreased* | 33 | 44 |
| Pleural effusion | 0 | 37 |
| Cellulitis | 1 | 32 |

^1^*The majority of neutropenia reports (97%) were from combination studies where subjects received lapatinib or blinded study medication in combination with chemotherapeutic agents such as paclitaxel, carboplatin, cisplatin, irinotecan or fluorouracil, all of which have been linked with neutropenia. In particular, 73% of neutropenia events occurred in patients participating in the paclitaxel combination study EGF30001.*

Overall, 30% of the SAEs reported were assessed as related to investigational product by the investigator. The ten most frequently reported drug related SAEs are listed in the table below:

| **MeDRA PT** | **Drug Related** | **Total (All Causalities)** |
| --- | --- | --- |
| Diarrhea* | 96 | 121 |
| Neutropenia | 39 | 48 |
| Vomiting* | 36 | 77 |
| Ejection fraction decreased* | 33 | 44 |
| Nausea* | 23 | 47 |
| Dehydration* | 21 | 49 |
| Febrile neutropenia | 18 | 25 |
| Mucosal inflammation | 13 | 16 |
| Pyrexia | 11 | 54 |
| Anemia | 10 | 28 |
| Ventricular dysfunction | 10 | 11 |

The most frequently reported drug related events were: diarrhea, neutropenia, vomiting, decreased ejection fraction, nausea and dehydration. Diarrhea which may lead to dehydration, nausea, vomiting and decreased ejection fraction are all included in the development core safety information (DCSI) for lapatinib. The majority of neutropenia/febrile neutropenia events (97%) were from combination studies where subjects received lapatinib or blinded study medication in combination with chemotherapeutic agents such as paclitaxel, carboplatin, cisplatin, irinotecan or fluorouracil, all of which have been linked with the event of neutropenia. In particular, 73% of neutropenia/febrile neutropenia events occurred in patients participating in the paclitaxel combination study EGF30001 (27).

**Information on Deaths**

As of 03 April 2006 112 subjects have died due to events reported as SAEs. Twenty of these deaths were assessed as related to investigational product by the investigator. Nine subjects received lapatinib monotherapy: gastrointestinal hemorrhage; sudden death; hepatic failure/hepatorenal syndrome/bacterial peritonitis; suture rupture; cerebral hemorrhage; renal/respiratory/cardiac failure; interstitial lung disease; jaundice; and dyspnea/cyanosis/pyrexia. Six subjects received lapatinib in combination with other chemotherapy: general physical health deterioration; neutropenic sepsis; septic shock; hepatic encephalopathy; cerebrovascular disorder/CNS metastases; upper gastrointestinal hemorrhage. Three subjects received capecitabine, one subject received placebo and one subject remains blinded (event was reported as related to combination therapy and unrelated to investigational product).

In addition there was one death for which an investigator relationship was not reported: this subject received lapatinib monotherapy and died due to an unspecified cause.

There is no overall pattern to these events and it should be noted that many subjects had concurrent medical history or were receiving concomitant medications which may have contributed to the event.

**Information on pregnancy**

As of 03 April 2006 there have been three pregnancy reports within the lapatinib clinical program. Study EGF10004 (27) subject 402 was found to be pregnant at an unknown time after the first dose of lapatinib. The subject gave birth to a normal female baby at 36 weeks gestation by caesarean section. Study EGF20002 (27) subject 2655 was found to be pregnant at an unknown time after receiving lapatinib and zoledronic acid. Treatment with lapatinib was discontinued. The patient experienced a spontaneous/missed abortion at 9 weeks gestational age which was reported as related to lapatinib by the investigator. Another subject in study EGF100642 (27) subject 101 became pregnant in the first month following her last dose of lapatinib. She later underwent an elective abortion due to progression of her underlying cancer.

**Cardiac and pulmonary toxicity**

Potential risks for the lapatinib program include cardiac toxicity and pulmonary toxicity which are known risks associated with other ErbB1 or ErbB2 inhibitors. Cardiac and pulmonary events are monitored closely in all lapatinib clinical trials and reviewed regularly by GSK. An Independent Data Monitoring Committee (IDMC) comprising oncologists, a cardiologist and a pulmonologist meet quarterly to assess the benefit-risk ratio in ongoing trials. Cardiac safety and interstitial pneumonitis are reviewed as part of this responsibility.

**Decreased Left Ventricular Ejection Fraction (LVEF)**

Left Ventricular Ejection Fraction (LVEF) has been evaluated using MUGA scans or echocardiogram during lapatinib phase I, II and III trials. As of 03 April 2006 a total of 61 subjects on the lapatinib program have experienced 63 events of decreased LVEF, regardless of investigational product received.

Of these 63 events, 57 events (55 subjects) met the protocol specific serious definition included in lapatinib phase II and III protocols:

- NCI CTC Grade 3 or 4 left ventricular systolic dysfunction *or*
- LVEF decrease ≥20% relative to baseline value *and* below the institutions lower limit of normal

Age range for subjects experiencing decreased LVEF was 31 to 81 years, with a median of 59 years. Seventy three percent of the reports were for female subjects, which may be attributed to the fact that the majority of large studies in the lapatinib program are for breast cancer. Sixty percent of the decreased LVEF events occurred within nine weeks of treatment onset. In 37 subjects, (67%) the event resolved or improved.

Forty one of the 55 subjects whose LVEF decrease met the protocol specific serious definition are known to have received lapatinib, giving an incidence of 1.3%. For the remaining 13 subjects: 6 remain blinded, 4 occurred on placebo, and 4 subjects received comparator (capecitabine or medroxy-progesterone) only. Of the 41 subjects on lapatinib, 32 LVEF decreases were assessed as related to investigational product by the investigator. Twenty three subjects (56%) were participating in monotherapy studies. Eighteen subjects received lapatinib in combination (3 capecitabine, 6 letrozole, 6 paclitaxel, 2 cisplatin, and 1 trastuzumab).

Of the 41 lapatinib subjects, 36 experienced asymptomatic LVEF decreases. Nineteen of these subjects were receiving lapatinib monotherapy, 17 subjects were receiving lapatinib in combination (capecitabine, letrozole, paclitaxel, or trastuzumab). In two of the 36 reports the subjects had no contributing factors and the events were ongoing at the time of the subject’s death due to disease progression. The remaining 34 subjects experienced asymptomatic LVEF decreases which were complicated by pre-existing conditions and/or previous/concurrent medications. Examples included: previous episodes of decreased LVEF, myocardial infarction, arrhythmia, left-chest radiation, and exposure to anthracyclines, trastuzumab, or paclitaxel, all of which have been associated with cardiac adverse events. In six of these 34 subjects the LVEF decrease resolved or improved on discontinuation of lapatinib (positive dechallenge).

Four lapatinib subjects experienced symptomatic LVEF decreases whilst receiving lapatinib monotherapy. The symptoms observed were dyspnea and cardiac failure, or palpitations. Three reports were complicated by pre-existing conditions and/or previous/concurrent medications which could have contributed to the event reported. The fourth subject had no contributing factors. All four events resolved promptly on discontinuation of lapatinib.

Follow-up is ongoing for the remaining lapatinib subject who experienced decreased LVEF. Current information indicates that this event was resolved after a 2 day duration; however it is unclear whether this event was symptomatic and why LVEF was measured on two consecutive days.

Many of the subjects in the lapatinib trials had previously received treatment with either anthracyclines and/or trastuzumab, both of which have been associated with cardiotoxicity. In addition some ongoing studies combine lapatinib with paclitaxel or capecitabine, both of which are associated with cardiotoxicity. This therefore increases the risk of cardiotoxicity in the lapatinib patients irrespective of any cardiac toxicity attributed to lapatinib. Decreased ejection fraction has been included in the DCSI for lapatinib.

**Interstitial Pneumonitis**

Current phase II and III lapatinib protocols include specific requirements for reporting of signs or symptoms of pneumonitis: subjects who have pulmonary symptoms which are NCI CTC Grade 3 or greater will be removed from study and the events documented as an SAE. As of 03 April 2006, 9 pulmonary events have been reported: 5 subjects experienced interstitial lung disease, 4 subjects experienced pneumonitis. Given the current figure of 3147 lapatinib subjects, this gives an approximate incidence of 0.25% for ‘pneumonitis type’ events. Seven of these events were reported as related to investigational product. Five subjects received lapatinib in combination, and three reports were from lapatinib monotherapy studies. The remaining subject did not receive lapatinib.

Age range for subjects experiencing signs or symptoms of pneumonitis was 48 to 73 years, with a median of 66 years. The majority of the reports (6/9) were for female subjects which may be attributed to the fact that there are a large number of breast cancer subjects in the lapatinib program. Five subjects (55%) who experienced pulmonary events recovered. Three of these 5 subjects required treatment, commonly methylprednisolone or prednisolone. For the remaining four subjects: two events were fatal, one event was ongoing at the time of the subject’s death due to disease progression, and one event was ongoing at the time of reporting. Both subjects who died were participating in monotherapy studies. The first subject died due to interstitial lung disease which the investigator attributed to disease progression. The second subject developed interstitial lung disease and died approximately seven months after the last dose of lapatinib. The subject had recently completed radiotherapy and the investigator felt that lapatinib may have affected the subject’s tolerance to radiotherapy.

Reports of signs/symptoms of pneumonitis are closely monitored and current data do not support the addition of this term to DCSI for lapatinib

**1.3.3.3 Biomarkers**

A Phase 1B trial evaluating tolerability and effects on biomarkers in tumor biopsies in heavily pre-treated patients with metastatic disease expressing EGFR or erbB2 reported that lapatinib was well tolerated at all dose levels tested (500-1600 mg/day) (30). Of 33 evaluable patients, 3 partial responses (PR) (10%) and 12 SD (36%) were reported. An evaluation of tumor biopsies obtained prior to, and after 2-3 weeks of treatment with lapatinib revealed that induction of apoptosis in tumor tissue and inhibition of intratumoral expression of activated Erk1/2, Akt and cyclin D protein appeared to correlate with PR and SD (30). Disease progression was associated with persistent, high levels of expression of these biomarkers. Treatment with lapatinib was not effective when insulin growth factor receptor (IGFR) was highly expressed (31). Tumor types in which objective response or stable disease were seen included: breast (7), colorectal (2), ovarian (2), lung (1), granular cell (1), and head and neck (1) carcinomas as well as adenocarcinoma of unknown primary (1). Objective responses were observed in ErbB2-expressing breast cancer patients who had progressed after receiving prior trastuzumab (Herceptin™)-based regimens. The most common adverse events included grade 1-2 rash (25%), diarrhea (27%), and nausea/vomiting (21%).

**1.3.4 Clinical Pharmacokinetics**

The pharmacokinetics of lapatinib are similar in healthy volunteers and patients, demonstrating oral absorption that is incomplete, highly variable, and sometimes delayed. After dosing, plasma concentrations rise to a peak at approximately 4 h and thereafter decline with measured half-lives averaging up to 14 h. However, accumulation with daily dosing achieves steady state in 6-7 days, which suggests a true elimination half-life on the order of 24 h. Administration of the same daily dose in a BD schedule results in 2‑fold greater systemic exposure than a OD schedule. Despite this inconsistency, systemic exposure generally increases with increasing dose. Absorption is increased by ingestion with food. Elimination of lapatinib is predominantly through metabolism by CYP3A4/5 with negligible renal excretion. Significant changes in systemic exposure to lapatinib result from co-administration of drugs that are potent inhibitors or inducers of CYP3A.

**1.4 Rationale for this Study**

HER2 (ErbB2), a member of the ErbB2 family of receptors, is expressed in 15-30% of primary breast cancers and its expression is associated with aggressive biological behaviour and poor disease outcome (22, 23). Breast cancer has been also shown to express high levels of EGFR and hormone-resistant disease is associated with an increased expression of both EGFR and EGFR ligands (32).

CTCs are identified in a significant proportion of patients with breast cancer both in early and metastatic disease (12, 16). The initial detection as well as the persistence of CTCs after the completion of systemic adjuvant treatment is associated with worse prognosis (12-14). Similarly, in the metastatic setting, the level of CTCs soon after the initiation of systemic treatment is a poor prognostic indicator (16). CTCs have been identified as malignant cells and at least theoretically bear the potential of giving rise to overt metastatic disease. In line with this, targeted-therapies such as monoclonal antibodies, have been proposed as alternative modalities capable of eliminating micrometastatic tumour cells resistant to prior chemotherapy or hormonal therapy (33).

HER2 expression has been demonstrated in occult tumor cells present in the bone marrow and/or the systemic circulation in 60% of breast cancer patients (34, 35). The expression of HER2 in CTCs is an independent prognostic factor for reduced survival (35). EGFR expression has been also demonstrated in circulating breast cancer cells by RT-PCR (36). The expression of both HER-2 and EGFR on CTCs has been confirmed by immunofluorescence microscopy (37). Both EGFR and HER-2 receptors influence many aspects of tumour cell biology including growth, survival, metastasis and angiogenesis. Selective inhibition of these growth factor receptors located on micrometastatic cells could theoretically interrupt the cellular signal transduction cascade leading to cell survival and subsequent development of metastatic disease.

The principal mechanism by which HER2 transmits signals is the formation of heterodimeric receptor complexes with EGFR, HER-3 or HER-4. It has been suggested that HER-2, being the most common heterodimerization partner of EGFR, may play an important role in the oncogenic activity of EGFR. In turn, EGF stimulation of EGFR, leads to activation of HER2 by heterodimerization (38). Thus, combined inhibition of both EGFR and HER2 may be more efficacious than targeting either one of them alone. Lapatinib is a selective reversible inhibitor of both EGFR and HER2 tyrosine kinases that inhibits receptor phosphorylation and downstream PI3K/Akt and MAP kinase signalling pathway in cells overexpressing EGFR or HER-2 (24, 25). Lapatinib has been shown to exert both antiproliferative and pro-apoptotic effects against breast cancer and represents an ideal candidate to be used for the elimination of circulating breast cancer cells. The feasibility of this approach has been demonstrated by the administration of trastuzumab in patients with breast cancer that resulted in the elimination of circulating cells in 22 out of 30 patients (33).

A significant amount of data point to the presence of CTCs in the peripheral blood of patients with solid malignancies as a prognostic indicator of poor clinical outcome (39). However, besides the clear prognostic value of CTCs, it is not yet known whether additional treatment targeting CTCs that persist despite systemic treatment, will translate into a benefit in progression-free and/or overall patient survival. At least theoretically, patients could benefit from a secondary treatment aiming in eliminating micrometastatic disease. This treatment could be tailored on the basis of the molecular profile of CTCs which reflect the subset of tumor cells that is potentially capable of giving rise to overt metastatic disease. This approach could be applicable either in the adjuvant and/or the metastatic disease setting.

Lapatinib is considered a novel and promising agent in the treatment of human breast cancer. Based on the above data, it could be very important and clinically relevant to know if lapatinib is capable of eliminating occult tumour cells circulating in the blood of breast cancer patients.

# 2. Study objectives

In the present study we would like to evaluate the efficacy of lapatinib in eliminating chemo- and/or hormone- resistant tumour cells circulating in the blood of patients with breast cancer.

- 1. **Primary objective**

The efficacy of lapatinib on HER2-positive CTCs will be measured by quantitative analysis of circulating tumour cells in the blood before, during and after the completion of lapatinib treatment. These cells will be detected by immunofluorescence microscopy on cytospins prepared from peripheral blood mononuclear cells (PBMCs) isolated by ficoll-hypaque density gradient centrifugation.

**2.2 Secondary objectives**

# 1. Correlation of the levels of CTCs with response to treatment

2. Assessment of the safety of lapatinib administration in this patient population

2.3. Exploratory Objectives

- Detection of cytokeratin-positive cells by immunofluorescence microscopy
- Detection of cytokeratin-positive/EGFR- positive CTCs (immunofluorescence microscopy)

# 3. Study plan and procedures

Patients will receive lapatinib monotherapy 1500 mg/day for a period of at least 1 month (unless disease progression occurs at an earlier time point). Blood samples will be collected at study entry, prior to the first dose and subsequently every month. Tumor assessment by imaging studies will be performed prior to study entry and subsequently every three months. At the end of the first month period patients with will be assessed for the presence of CTCs in their peripheral blood. Patients with decreased or stable CTC counts will continue treatment and will be assessed every month. Patients with increased CTC counts will be withdrawn from the study if further increase is documented at re-assessment after the second treatment course. In subsequent cycles, treatment will be also discontinued in case of two consecutive increases in CTC values. Moreover, treatment will be discontinued in case of disease progression, occurrence of unacceptable toxicity or consent withdrawal, irrespectively of CTC counts.

##

## 3.1 Overall study design and flow chart

Patients will receive lapatinib monotherapy 1500 mg/day for a period of at least one month depending on their response and the clinical status of their disease.

- In this pilot study 20 women with metastatic breast cancer who have circulating occult tumour cells in the blood (defined as the presence of ≥ 1 CTC/ 10^6^ PBMCs detected by immunofluorescence microscopy) despite previous chemotherapy and hormone therapy will be treated with lapatinib. All patients will be enrolled in the Medical Oncology Unit at the University Hospital of Heraklion
- All patients will receive lapatinib for a minimum of one month unless clinical disease progression (defined by RECIST criteria) occurs at an earlier time point. At the end of each month period patients will be assessed for the presence of CTCs and treatment will be administered accordingly as described above.
- Open label trial
- Non randomised
- Primary outcome variable: effect of lapatinib on tumor cells circulating in the blood of breast cancer patients
- Target patient population: Patients with metastatic breast cancer who have been previously treated with chemotherapy and/ or hormonal therapy
- Treatment with lapatinib monotherapy 1500 mg/day

Study flow chart is presented in Table 4.

## Table 4. Study design and flow chart

| **SCREENING PERIOD DAY -21 to 0**  Patient informed consent; past medical history; demography; tumour assessment (chest X‑ray, computerised tomography [CT] scan and bone scan), CTCs assessment (double staining immunofluorescence microscopy for HER2/cytokeratin) |
| --- |
|  |
| **MAIN TREATMENT PERIOD (1 MONTH)**  Patients evaluated for concurrent illness/therapy; physical examination, adverse events at all visits (every month)  Every month: haematology; biochemistry; tumour assessment, CTCs assessment (double staining immunofluorescence experiments)  At 3 months (or at any time in case of suspected clinical disease progression): chest X‑ray, computerised tomography [CT] scan and bone scan) |
|  |
| **SECOND TREATMENT PERIOD**  Patients evaluated for concurrent illness/therapy; physical examination, adverse events at all visits (every month)  Every month: haematology; biochemistry; tumour assessment, CTCs assessment (double staining immunofluorescence experiments)  Every 3 months (or at any time in case of suspected clinical disease progression): chest X‑ray, computerised tomography [CT] scan and bone scan)  . |

Table 5 STUDY PLAN

| Study plan | Screening | Start of treatment | Treatment | | | | | | Discontinuation |
| --- | --- | --- | --- | --- | --- | --- | --- | --- | --- |
| Day (or week) | -21 to  -7 | **0** | +4we | +8we | +12we | +16 we | +20 we | +24 we |  |
| Visit | V1 | V2 | V3 | V4 | V5 | V6 | V7 | V8 |  |
| Informed consent | X |  |  |  |  |  |  |  |  |
| Medical history | X |  |  |  |  |  |  |  |  |
| Pregnancy test (if appropriate) |  | X^a^ |  |  |  |  |  |  |  |
| Inclusion/exclusion criteria | X | X |  |  |  |  |  |  |  |
| Physical examination including Vital signs^b^ / Performance Status / weight |  | X | X | X | X | X | X | X | X |
| Concurrent medication |  | X^c^ | X | X | X | X | X | X | X |
| ECG |  | X |  |  |  |  |  |  |  |
| Clinical chemistry/ Hematology^b^ |  | X | X | X | X | X | X | X |  |
| Tumour assessment | X |  |  |  | X |  |  | X |  |
| CTCs assessment (CTCs number, double staining immunofluorescence microscopy | X | X | X | X | X | X | X | X | X |
| Adverse events^c^ |  |  | X | X | X | X | X | X | X^,de^ |
| Dispense study medication |  | X | X |  |  | X |  |  |  |
| Survival |  |  |  |  |  |  |  |  | X^f^ |

a Pre-menopausal women of child-bearing potential must have a negative urine or serum pregnancy test within 7 days before Day 1 of treatment, In the event of suspected pregnancy during the study, the test should be repeated.

b Laboratory/vital signs abnormalities should not be reported as adverse events (AEs) unless any criterion for a serious adverse event (SAE) is fulfilled, or laboratory/vital signs abnormality causes the subject to discontinue from the study, or the investigator insists the abnormality should be reported as an adverse event.

c In the event of any new or worsening respiratory symptoms (e.g. cough, breathlessness), appropriate medical advice should be sought promptly. Any symptoms should be treated as clinically indicated and reported as an AE or SAE if appropriate.

d Any ongoing study-related toxicity or SAE at discontinuation must be monitored until resolution.

e After discontinuation from treatment, subjects must be followed up for all existing and new AEs for 30 calendar days after the last dose of study drug. All new AEs occurring during that period must be recorded (if SAEs, they must be reported within 24 hours) and followed to resolution where possible.

f After discontinuation from the study for any reason, where possible subject, subject’s family, or the subject’s current physician must be contacted every 8 weeks for survival information until death.

## 3.2 Rationale for study design and dose

Based on preclinical and clinical data, lapatinib is considered a novel and promising therapeutic approach with potential application in the treatment of human breast cancer. Therefore it could be very important and clinically relevant to know if lapatinib is capable of reducing the numbers of occult tumour cells circulating in the blood of breast cancer patients.

Lapatinib will be given at the dose of 1500mg/day for a minimum of 3 months. Results from phase 1 studies support the use of 1500 mg po daily as achieving biologically active concentrations and target pathway inhibition with an acceptable toxicity profile. Phase II studies of lapatinib administered as monotherapy demonstrated that the dose of 1500mg/day was well tolerated and was associated with clinical benefit in patients with breast cancer (27, 29).

The selected patient population is patients with metastatic breast cancer who have received at least one course of standard systemic chemotherapy for their metastatic disease. Prior hormonotherapy is allowed.

## 3.3 Selection of study population

### 3.3.1 Inclusion criteria

For inclusion in the study subject must fulfil all of the following criteria:

1. .Provision of written informed consent
2. Histologically or cytologically‑confirmed breast cancer
3. Metastatic breast cancer (stage IIIB and IV). EGFR and/or HER-2 expression on the primary tumor is not mandatory.
4. Patients should have received at least one course of standard systemic chemotherapy for their metastatic disease. Prior hormonal therapy is allowed.
5. Patients should have achieved objective response (CR or PR) or stable disease to previous treatment.
6. There should be at least one month between the end of chemotherapy treatment and trial entry. In case of prior Herceptin administration, 3 months are required to have elapsed before study entry.
7. Detection of at least 1 CTC/10^6^ PBMCs despite the previous administration of chemotherapy and/or hormonal therapy.
8. HER-2 expression on CTCs.
9. Age 18 years and over Adequate organ function

Haematological function:

- Absolute neutrophil count (ANC) ≥1.5 x 10^9^/L
- Platelet count ≥100 x 10^9^/L and
- Haemoglobin ≥9 g/dL (may be transfused to maintain or exceed this level)

Liver Function:

- Total bilirubin <1.5 x upper limit of normal (ULN)
- AST, ALT <2.5 x ULN in patients without liver metastases; <5 x ULN in patients with liver metastases

Renal function:

- Serum creatinine ≤1.25 x ULN or calculated creatinine clearance ≥50 mL/min

1. LVEF within institutional normal range
2. Eastern Cooperative Oncology Group (ECOG) performance status (PS) of 0 to 2
3. Life expectancy of at least 12 weeks

###

### 3.3.2 Exclusion criteria

Any of the following is regarded as a criterion for exclusion from the study:

1. any concurrent systemic treatment for breast cancer (including chemotherapy, radiotherapy, monoclonal antibodies)
2. Other co-existing malignancies or malignancies diagnosed within the last 5 years with the exception of basal cell carcinoma or cervical cancer in situ
3. Any unresolved chronic toxicity greater than Grade 2 (NCI- CTCAE) from previous anticancer therapy (except alopecia)
4. Any evidence of severe or uncontrolled systemic disease (e.g., unstable or uncompensated respiratory, cardiac, hepatic, or renal disease)
5. Pregnancy or breast feeding (women of child-bearing potential). Women of childbearing potential must practice acceptable methods of birth control to prevent pregnancy
6. Treatment with any other investigational agent, or participation in another clinical trial within 28 days prior to enrolment
7. Known hypersensitivity to drugs chemically related to lapatinib
8. Concomitant requirement for medication classified as CYP3A4 inducers or inhibitors.
9. Evidence of any other disease, neurological or metabolic dysfunction, physical examination finding or laboratory finding giving reasonable suspicion of a disease or condition that contraindicates the use of an investigational drug or puts the patient at high risk for treatment-related complications

###

### 3.3.3 Discontinuation of subjects from treatment or assessment

#### 3.3.3.1 Criteria for Discontinuation

Subjects may be discontinued from study treatment and assessments at any time. Specific reasons for discontinuing a subject from this study are:

1. voluntary discontinuation by the subject who is at any time free to discontinue their participation in the study, without prejudice to further treatment.
2. safety reasons as judged by the investigator
3. severe non-compliance to the protocol as judged by the investigator
4. incorrect enrolment of the subject*.*
5. death
6. subject lost to follow-up
7. clinical progression of disease (by symptoms, physical findings, imaging studies)
8. patients with two concecutive increases in CTC numbers

#### 3.3.3.2 Procedures for discontinuation

#### Subjects who discontinue should, if possible, be seen and assessed by an investigator(s). The reason for discontinuation and the date of discontinuation must be documented on the case report form (CRF). If possible any diary cards, questionnaires (e.g. for Quality of Life assessments) and investigational products should be returned by the subject.

All subjects who have new or worsening CTC grade 3 or 4 laboratory values at the time of discontinuation must have further tests performed and the results recorded on the appropriate CRF until the laboratory values have returned to CTC grade 1 or 2, unless these values are not likely to improve because of the underlying disease. In these cases, the investigators must record their opinions on the CRFs and in the subject’s medical records. Laboratory abnormalities should not be reported as adverse events unless any criterion for an SAE is fulfilled, the laboratory abnormality causes the subject to discontinue from the study, or the investigator insists the abnormality should be reported as an AE.

At discontinuation all on-going study-related toxicities and SAEs must be followed until resolution, unless in the investigator’s opinion, the condition is unlikely to resolve due to the subject’s underlying disease.

After discontinuation from treatment, subjects must be followed up for all existing and new AEs for 30 calendar days after the last dose of study drug. All new AEs occurring during that period must be recorded (if SAEs they must be reported within 24 hours) and followed up for resolution as above.

Any subject who withdraws from study treatment for reasons other than progression should continue, where possible, to have objective tumour assessments every 8 weeks in order to collect information on progression of disease.

When disease progression has been documented, the long-term follow up information for survival should be collected at least every 8 weeks by telephone contact with the subject, subject’s family, or by contact with the subject’s current physician.

## 3.4 Treatments

### 3.4.1 Investigational products

(a) Lapatinib

GlaxoSmithKline will supply Lapatinib to the investigator for use

#### Descriptive information for Lapatinib can be found in the Investigator’s Brochure

####

#### 3.4.1.1 Doses and treatment regimens

1. Lapatinib

Lapatinib is supplied as 250 mg oval, biconvex, orange film-coated tablets. The lapatinib dose level for this study is 1500 mg. The tablets are packaged into HDPE bottles with child-resistant closures. Study treatment will be dispensed to subjects on Day 1 and every monthly visit thereafter until the subject withdraws, or completes the study.

**Route of administration**

Lapatinib treatment will be taken orally once a day, every day about the same time. Patients should be advised to take lapatinib on an empty stomach (either 1 hour before or 1 hour after meals). If the subject forgets to take a dose, they should take the last missed dose as soon as they remember, as long as it is at least 12 hours before the next dose is due.

#### Guideline for dispersing whole lapatinib tablets

The following procedure is recommended for administering dispersed whole tablet(s) to patients who are unable to swallow:

Place ½ cup (120mL) of water in a glass container, then add six 250 mg lapatinib tablets to the container. Cover the container, let it stand for 5 minutes, and then stir the mixture intermittently for 10-20 minutes or until it is fully dispersed. Stir the container for 5 seconds then administer. Rinse the container with a ¼ cup (60mL) aliquot of water and administer (total of ¾ cup, 180mL of liquid is dispensed).

1. Lapatinib dose interruption/reduction

See section 3.5 - Management of Toxicity

####

#### 3.4.1.2 Storage

The intact bottles should be stored at controlled room temperature (15°C-30°C).

#### 3.4.1.3 Accountability

- Deliveries of such products from GlaxoSmithKline are correctly received by a responsible person (e.g., a pharmacist)
- Deliveries are recorded
- Study treatments are handled and stored safely and properly
- Study treatments are dispensed only to study subjects in accordance with the protocol
- Subjects must return all unused medication and empty containers to the investigator

At the end of the study, it must be possible to reconcile delivery records with records of usage and destroyed/returned stock. Any discrepancies must be accounted for. Certificates of delivery and return must be signed, preferably by the investigator or a pharmacist.

The principal investigator/sub-investigator must request the subject to return unused drugs which may be left over for subject’s negligence or any other reasons, as well as empty cartons and packaging

### 3.4.2 Pre-study, concomitant and post-study treatment(s)

#### 3.4.2.1 Treatment for cancer

Except for lapatinib, no systemic treatment or radiotherapy known to have an effect on breast cancer may be used during the study.

####

#### 3.4.2.2 Lapatinib Prohibited Medication List

Gastric pH Modifiers. GSK is no longer prohibiting gastric pH modifiers (i.e. H2 blockers and PPIs). This is based on collection of concurrent med use and the observation that PK was no different between pts on gastric pH modifiers and those not taking gastric pH modifiers.

Lapatinib is a substrate for CYP3A4. Inducers and inhibitors of CYP3A4 may alter the metabolism of lapatinib. The following list of CYP3A4 inducers and inhibitors are prohibited from screening through discontinuation from study.

| **Drug Class** | **Agent** | **Wash-out^1^** |
| --- | --- | --- |
| **CYP3A4 Inducers** | | |
| Antibiotics | all rifamycin class agents (e.g., rifampicin, rifabutin, rifapentine) | 14 days |
| Anticonvulsants | phenytoin, carbamezepine, barbiturates (e.g., phenobarbital) |  |
| Antiretrovirals | efavirenz, nevirapine |  |
| Glucocorticoids (oral) | cortisone (>50 mg), hydrocortisone (>40 mg), prednisone (>10 mg), methylprednisolone (>8 mg), dexamethasone (>1.5 mg)^2^ |  |
| Other | St. John’s Wort, modafinil |  |
| **CYP3A4 Inhibitors** | | |
| Antibiotics | clarithromycin, erythromycin, troleandomycin | 7 days |
| Antifungals | itraconazole, ketoconazole, fluconazole (>150 mg daily), voriconazole |  |
| Antiretrovirals, Protease Inhibitors | delaviridine, nelfinavir, amprenavir, ritonavir, indinavir, saquinavir, lopinavir |  |
| Calcium channel blockers | verapamil, diltiazem |  |
| Antidepressants | nefazodone, fluvoxamine |  |
| GI Agents | cimetidine, aprepitant |  |
| Other | grapefruit, grapefruit juice |  |
|  | amiodarone | 6 months |
| **Miscellaneous** | | |
| Antacids | Mylanta, Maalox, Tums, Rennies | Excluded 1 hour before and after dosing |
| Herbal or dietary supplements | ginkgo biloba, kava, grape seed, valerian, ginseng, echinacea, evening primrose oil, and St John's Wort | 14 days |
| 1. At the time of screening, if a patient is receiving any of the above listed medications/substances, the medication or substance must be discontinued (if clinically appropriate) for the period of time specified prior to administration of the first dose of lapatinib and throughout the study period in order for the patient to be eligible to participate in the study. 2. Glucocorticoid daily doses (oral) ≤ 1.5 mg dexamethasone (or equivalent) are allowed. Glucocorticoid conversions are provided in parentheses. | | |

## 3.5 Management of Toxicity

**3.5.1 Criteria for Evaluating Cardiac and Respiratory Events**

**3.5.1.1 Asymptomatic Events**

Subjects who have a ≥ 20% decrease in left ventricular cardiac ejection fraction (LVEF) relative to baseline, and the LVEF is below the institution's lower limit of normal, should have a repeat evaluation of ejection fraction 1-2 weeks later while still receiving investigational product.

If the repeat ejection fraction evaluation confirms a ≥ 20% decrease in LVEF relative to baseline, and the ejection fraction is below the institution's lower limit of normal, then investigational product should be temporarily discontinued.

If the LVEF recovers during the next 3 weeks, after consultation and approval by the GSK Medical Monitor, the subject may be restarted on study treatment at a reduced dose. For such subjects, monitoring of LVEF will then be performed 2 weeks and 4 weeks after re-challenge, and then every 4 weeks thereafter.

If repeat ejection fraction evaluation still shows a decrease ≥ 20% in LVEF relative to baseline and the value is below the institution's lower limit of normal, then the subject should be withdrawn from investigational product. Ejection fraction should continue to be monitored every 4 weeks for at least 16 weeks or until resolution.

**3.5.1.2 Symptomatic cardiac events**

**Subjects with an NCI CTCAE Grade 3 or 4 left ventricular systolic dysfunction or interstitial pneumonitis must be withdrawn from therapy.**

Note: Copies of echocardiograms and/or MUGA scans performed on subjects who experience a ≥ 20% decrease in LVEF relative to baseline (asymptomatic or symptomatic), and a cardiac ejection fraction is below the institution's lower limit of normal, will be required by GSK for review by an independent board. A ≥ 20% relative decrease from baseline in LVEF (asymptomatic or symptomatic), that is below the institution’s lower limit of normal is considered an SAE and must be reported to GSK.

**3.5.2 Rash**

Skin rash (usually grade 1-2) has been observed during the first several days of treatment with EGFR inhibitors and has been observed to diminish in severity after 4 weeks of treatment in many patients. In some patients, this rash appeared to be treatable with standard acne therapies, including topical and oral antibiotics used to treat acne. Anecdotal reports of improvement have occurred with several agents. In patients with severe rash, treatment may need to be discontinued or the dose reduced. Anecdotal reports of improvement have occurred with any of the following: minocycline, topical tetracycline, topical clindamycin, topical silver sulfadiazine, diphenhydramine, oral prednisone (short course).

**3.5.3** **Diarrhea**

Diarrhea has been seen with lapatinib and with other EGFR inhibitors. In general EGFR inhibitor-induced diarrhea has been transient, usually not of sufficient severity to hinder administration of the agents, and responsive to loperamide. The recommended dose of loperamide is 4 mg at first onset, followed by 2 mg q 2–4 hr until diarrhea free for 12 hr.

**Lapatinib Diarrhea Management Guidelines:**

1. Uncomplicated grade 1-2 diarrhea:
   - Stop all lactose containing products;
   - Drink 8-10 large glasses of clear liquids a day;
   - Eat frequent small meals;
   - Grade 2 diarrhea hold cytotoxic chemotherapy;
   - Grade 2 diarrhea consider dose reduction of lapatinib (discuss with medical monitor);
   - Administer standard dose of loperamide:
     - Initial dose 4mg followed by 2mg every 4 hours or after every unformed stool.
     - We suggest continuation of loperamide until diarrhea free for 12 hours.
2. For grade 3 or 4 diarrhea or grade 1 or 2 with complicating features (severe cramping, severe nausea/vomiting, decreased performance status, fever, sepsis, grade 3 or 4 neutropenia, frank bleeding, dehydration):
   - Use intravenous fluids as appropriate, consider hospital admission;
   - Use prophylactic antibiotics as needed (example fluoroquinolones), especially if diarrhea is persistent beyond 24 hours or there is fever or grade 3-4 neutropenia;
   - Hold both cytotoxic chemotherapy and lapatinib and discuss with medical monitor.

These broad general management principles are recommended to proactively try and avoid more serious complications by active management of the diarrhea syndrome. Guidelines such as these should never replace sound clinical judgment. Our experience thus far suggests that when lapatinib is used as monotherapy we mostly are dealing with uncomplicated grade 1 or 2 diarrheas. These general management principles do not discuss comprehensive management of more serious or protracted diarrhea syndromes.

**3.5.4 Nausea**

Routine premedication for nausea is not necessary, but symptomatic patients should be treated with standard antinausea/antiemetic therapy as necessary.

If the patient vomits after taking the tablets, the dose is replaced only if the tablets can actually be seen and counted.

**3.6 Dosing delays/dose modifications**

Starting dose and dose modifications for unacceptable toxicity are listed in Table 6. For toxicity that is thought to be related to lapatinib, the daily dose of lapatinib will be decreased according to the schedule displayed in the Table 7.

Dose adjustments are to be made according to the greatest degree of toxicity. Toxicities will be graded using NCI Common Terminology Criteria for Adverse Events (CTCAE) version 3.0.

The most common toxic effects of lapatinib are:

**Metabolism and nutrition disorders:**

Anorexia

**Cardiac disorders:**

Decreased ejection fraction

**Gastrointestinal Disorders:**

Diarrhea which may lead to dehydration; nausea, and vomiting.

**Skin and subcutaneous tissue disorders:**

Rash (including dermatitis acneiform)

**General disorders and administration site conditions:**

Fatigue

**3.6.1 Dose Delays and Dose Reductions**

Treatment may be delayed, up to 2 weeks, to allow for resolution of toxicity except in the event of NCI CTCAE Grade 3 or 4 left ventricular cardiac dysfunction or NCI CTCAE Grade 3 or 4 interstitial pneumonitis. The Investigator must consult the GSK Medical Monitor prior to continuing therapy for any subject requiring a delay of more than 2 weeks for unresolved toxicity, but in general, such subjects should be withdrawn from the study. If treatment is delayed for reasons other than toxicity (i.e., unplanned travel or vacation, or lack of transportation to the site) and the subject has insufficient investigational product available, the subject should resume the usual dosing schedule once drug supply has been made available. However, if the subject has been off therapy for more than 2 weeks, the Investigator must consult the GSK Medical Monitor prior to continuing therapy.

Dose reduction for drug-related toxicity is permitted; however the GSK Medical Monitor must be consulted prior to implementing any change in dosing. Only one dose reduction is permitted per subject and subjects should not be rechallenged to a higher dose level. If a NCI CTCAE Grade 3 or 4 drug-related event (other than left ventricular cardiac dysfunction or interstitial pneumonitis) has occurred, the investigator may discuss with the GSK Medical Monitor whether a reduction of dose is appropriate.

**Table 6. Lapatinib Starting Dose and Dose Reduction Schedule**

| Starting dose | 1500 mg/day | *** Patients requiring a third dose reduction should be taken off study unless in the opinion of the investigator & GSK Medical Monitor, there is reason to believe that the patient is still experiencing clinical benefit |
| --- | --- | --- |
| First dose reduction | 1250 mg/day |

* if no recovery after 3 weeks of holding drug, patients should go off study unless in the opinion of the investigator & GSK Medical Monitor, there is reason to believe that the

patient is still experiencing clinical benefit.

**Table 7**. Dose Reduction Criteria and Guidelines for Management of lapatinib Associated Toxicity

|  | Grade | **Guideline for management** | **lapatinib dosage modification*** |
| --- | --- | --- | --- |
| Diarrhea | 1 | No intervention | None |
|  | 2 | Loperamide (4 mg at first onset,  followed by 2 mg every 2–4 hrs  until diarrhea free for 12 hrs) | None; If unacceptable to patient or medically concerning then hold until recovery  to < grade 1, up to 21 days*. Restart at same dose**. |
|  | > 3 (*despite optimal*  *use of loperamide)* |  | Hold until recovery to < grade 1, up to 21 days*. And then reduce 1 dose level. |
| Rash | 1 | No intervention | None |
|  | 2 | Any of the following:  minocycline^+^, topical  tetracycline or clindamycin,  topical silver sulfadiazine,  diphenhydramine,  oral prednisone (short course) | None; If unacceptable to patient or medically concerning then hold until recovery  to < grade 1, up to 21 days*. Restart at same dose**. |
|  | > 3 |  | Hold until recovery to < grade 1, up to 21 days*. And then reduce 1 dose level. |
| Other  Toxicity **(excluding**  **left ventricular**  **dysfunction and**  **pneumonitis,**  **see below)** | 1 | No Intervention | None |
|  | 2 | Treatment as appropriate | None; If unacceptable to patient or medically concerning then hold until  recovery to < grade 1, up to 21 days*. Restart at same dose **. |
|  | 2 prolonged or  clinically significant  and grade > 3 | Treatment as appropriate | Hold until recovery to < grade 1, up to 21 days and then reduce 1 dose level* |
| * if no recovery after 3 weeks of holding drug, patients should go off study unless in the opinion of the investigator & GSK Medical Monitor, there is reason to believe that the patient is still experiencing clinical benefit.  ** if dose has been previously held for grade 2 toxicity and grade 2 symptoms recur, OR if the patient finds the symptoms unacceptable, hold dose until recovery to < grade 1 and then reduce dose one level  + recommended dose: 200mg po bid (loading dose), followed by 100mg po bid for 7-10 days | | | |

**4. REGULATORY AND REPORTING REQUIREMENTS**

It is the responsibility of the investigator to document all adverse events which occur during the investigation. An adverse event is any untoward medical occurrence in a patient or clinical investigation subject, temporally associated with the use of the medicinal product, whether or not considered related to the medicinal product. *An AE can therefore be any unfavorable and unintended sign (including an abnormal laboratory finding), symptom, or disease (new or exacerbated) temporally associated with the use of a medicinal product.* Anticipated day-to-day fluctuations of the disease under study that do not represent a clinically significant exacerbation or worsening need not be considered an adverse event.

All adverse events occurring from the first dose of investigational product until five days after the last dose must be recorded **regardless of whether or not they are considered drug related.** In addition, any SAEs which occur as a result of protocol specific diagnostic procedures or interventions must also be reported.

**The sponsor is responsible under ICH GCP for reporting all safety events and information to the regulatory authorities and relevant ethics committee as required within the given reporting timelines.**

**4.1 Assessment of Causality**

Every effort should be made by the investigator to explain each adverse event and assess its relationship, if any, to study drug treatment. Causality should be assessed using the following categories: no (not related), or yes (reasonable possibility).

The degree of certainty with which an adverse experience is attributed to drug treatment (or alternative causes, e.g. natural history of the underlying diseases, concomitant therapy, etc.) will be determined by how well the experience can be understood in terms of the following:

**·** Known pharmacology of the drug

**·** Reaction of similar nature being previously observed with this drug or class of drug

**·** The event having often been reported in literature for similar drugs as drug related (e.g. skin rashes, blood dyscrasia)

**·** The event being related by time to drug administration terminating with drug withdrawal (dechallenge) or reproduced on rechallenge.

The investigator may change his/her opinion of causality in light of follow-up information, amending the SAE form. The causality assessment is one of the criteria used when determining regulatory reporting requirements.

## 4.2 Following-up of Adverse Events

Investigators should follow-up subjects with adverse events until the event has subsided (disappeared) or until the condition has stabilized.

### 4.3 Definition of Serious Adverse Events

A serious adverse experience is any event which is fatal, life threatening, disabling or incapacitating or results in hospitalization, prolongs a hospital stay or is associated with congenital abnormality. In addition, any experience which the investigator regards as serious or which would suggest any significant hazard, contraindication, side effect or precaution that may be associated with the use of the drug should be reported as a serious adverse event. Examples of such events are invasive or malignant cancers, intensive treatment in an emergency room or at home for allergic bronchospasm, blood dyscrasias or convulsions that do not result in hospitalization, or development of drug dependency or drug abuse.

Abnormal laboratory findings (e.g., clinical chemistry, hematology) or other abnormal

assessments (e.g., x-rays, scans, vital signs, etc.) that are judged by the investigator as

**clinically significant** will be recorded as AEs or SAEs if they meet the definition of an

AE or SAE.

**4.3.1 Life threatening definition**

An adverse event is life threatening if the patient was at immediate risk of death from the event as it occurred (i.e. it does not include a reaction that if it had occurred in a more serious form might have caused death). For example, drug-induced hepatitis that resolved without evidence of hepatic failure would not be considered life threatening even though drug-induced hepatitis could be fatal.

**4.3.2 Disability/incapacitating definition**

An adverse experience is incapacitating or disabling if the experience results in a substantial and/or permanent disruption of the patient's ability to carry out normal life functions.

**4.3.3 Hospitalization definition**

Hospitalization for elective treatment of a pre-existing condition that did not worsen from baseline is not considered an AE.

**4.3.4 Additional SAE definitions**

- All Grade 4 laboratory abnormalities

- Cardiovascular events have been seen in subjects taking other compounds that inhibit ErbB2 when used in combination with or following anthracyclines and interstitial pneumonitis has been reported in subjects taking compounds that inhibit ErbB1. As a precaution, the following will be reported as a SAE:

Cardiac dysfunction will be reported as an SAE and will be defined as any signs or symptoms of deterioration in left ventricular cardiac function that are Grade 3 (NCI CTCAE) or a **>**20% decrease in left ventricular cardiac ejection fraction relative to baseline, which is below the institution's lower limit of normal. Refer to NCI CTCAE grading of left ventricular cardiac function.

Subjects who have a **>**20% decrease in left ventricular cardiac ejection fraction relative to baseline, and the ejection fraction is below the institution's lower limit of normal, should have a repeat evaluation of ejection fraction 1-2 weeks later while still receiving investigational product (lapatinib). If the repeat ejection fraction evaluation confirms a **>**20% decrease in left ventricular cardiac ejection fraction, and the ejection fraction is below the institution's lower limit of normal, then investigational product should be temporarily discontinued. If the left ventricular ejection fraction recovers during the next 3 weeks, after consultation and approval of the medical monitor, the subject may be restarted on investigational product (lapatinib) at a reduced dose. For such subjects, monitoring of left ventricular ejection fraction will then be performed 2 weeks and 4 weeks after re-challenge and then every 4 weeks, thereafter. If repeat ejection fraction evaluation still shows a decrease **>**20% in left ventricular ejection fraction relative to baseline, and the value is below the institution's lower limit of normal, then the subject should be withdrawn from investigational product (lapatinib).

- Any signs or symptoms of pneumonitis that are >Grade 3 (NCI CTCAE) (defined as radiographic changes and requiring oxygen). Refer to NCI CTCAE grading of pneumonitis/pulmonary infiltrates.

### 4.4 Reporting Serious Adverse Events

Any serious adverse events which occur during the clinical study or within 30 days of receiving the last dose of study medication, whether or not related to the study drug, must be reported by the investigator. In addition, any SAEs which occur as a result of protocol specific diagnostic procedures or interventions must also be reported.

## 4.4.1 Lack of Efficacy

“Lack of efficacy” *per se* will not be reported as an AE. The signs and symptoms or clinical sequelae resulting from lack of efficacy will be reported if they fulfil the AE or SAE definition (including clarifications).

**All serious adverse events, in addition to being reported to the regulatory body and approving ethics committee by the investigator, must be reported by facsimile within 24 hours to GlaxoSmithKline.**

**Fax: <enter LOC fax number>**

**For medical emergencies contact:**

**< enter name of LOC physician> Office: <enter LOC office phone number>**

**or**

**<enter name of LOC scientist> Office: <enter LOC office phone number Mobile: <if required>**

**Toll Free Number: <enter local toll free number if available>**

**After Hours or Weekends: <enter out of hours number> ask for physician on call**

**GlaxoSmithKline**

**< enter LOC address>**

**< >**

**< >**

**< >**

The SAE report should comprise a full written summary, detailing relevant aspects of the adverse events in question. Where applicable, information from relevant hospital case records and autopsy reports should be included. Follow-up information should be forwarded to GSK within 24 hours.

SAEs brought to the attention of the investigator at any time after cessation of lapatinib and considered by the investigator to be related or possibly related to lapatinib must be reported to GSK if and when they occur. Additionally, in order to fulfil international reporting obligations, SAEs that are related to study participation (e.g., procedures, invasive tests, change from existing therapy) or are related to a concurrent medication will be collected and recorded from the time the subject consents to participate in the study until he/she is discharged.

## 4.5 Pregnancy

Patients who become pregnant during the study should discontinue the study immediately.

Patients should be instructed to notify the investigator if it is determined after completion of the study that they become pregnant either during the treatment phase of the study or within five days after the treatment period.

Whenever possible a pregnancy should be followed to term, any premature termination reported, and the status of the mother and child should be reported to GlaxoSmithKline after delivery.

**REFERENCES**

- - 1. Lambrechts AC, van Veer LJ, Rodenhuis S: The detection of minimal numbers of contaminating epithelial tumor cells in blood or bone marrow: Use, limitations and future of RNA-based methods. Ann Oncol 9: 1269-1276, 1998.
    2. Pantel K, Cote RJ, Fodstad O: Detection and clinical importance of micrometastatic disease. J Natl Cancer Inst 91: 1113-1124, 1999.
    3. Cote RJ, Rosen PP, Lesser ML, et al: Prediction of early relapse in patients with operable breast cancer by detection of occult bone marrow micrometastases. J Clin Oncol 9: 1749-1756, 1991.
    4. Braun S, Pantel K, Muller P, et al: Cytokeratin-positive cells in the bone marrow and survival of patients with stage I, II or III breast cancer. N Engl J Med 342: 525-533, 2000.
    5. Diel IJ, Kaufmann M, Costa SD, et al: Micrometastatic breast cancer cells in bone marrow at primary surgery: prognostic value in comparison with nodal status. J Natl Cancer Inst 88: 1652-1658, 1996.
    6. Mansi JL, Easton D, Berger U, et al: Bone marrow micrometastases in primary breast cancer: prognostic significance after 6 years’ follow up. Eur J Cancer 27: 1552-1555, 1991.
    7. Schoenfeld A, Kruger KH, Gomm J, et al: The detection of micrometastases in the peripheral blood and bone marrow of patients with breast cancer using immunohistochemistry and reverse transcriptase polymerase chain reaction for keratin 19. Eur J Cancer 33: 854-861, 1997.
    8. Datta YH, Adams PT, Drobyski WR, et al: Sensitive detection of occult breast cancer by the reverse-transcriptase polymerase chain reaction. J Clin Oncol 12: 475-482, 1994.
    9. Moscinski LC, Trudeau WL, Fields KK, et al: High-sensitivity detection of minimal residual breast carcinoma using the polymerase chain reaction and primers for cytokeratin-19. Diagn Mol Pathol 5: 173-180, 1995.
    10. Kruger W, Krzizanowski C, Holweg M, et al: Reverse transcriptase-polymerase chain reaction detection of cytokeratin-19 mRNA in bone marrow and blood of breast cancer patients. J Cancer Res Clin Oncol 122: 679-686, 1996.
    11. Stathopoulou A, Gizi A, Perraki M, et al: Real-time quantification of CK-19 mRNA-positive cells in peripheral blood of breast cancer patients using the lightcycler system. Clin Cancer Res 9: 5145-5151, 2003.
    12. Stathopoulou A, Vlachonikolis J, Mavroudis D, et al: Molecular detection of cytokeratin-19-positive cells in the peripheral blood of patients with operable breast cancer: Evaluation of their prognostic significance. J Clin Oncol 20: 3404-3412, 2002.
    13. Xenidis N, Perraki M, Kafousi M, et al: Predictive and prognostic value of peripheral blood cytokeratic-19 mRNA positive cells detected by real time PCR in node-negative breast cancer patients. J Clin Oncol 24: 3756-3762, 2006.
    14. Braun S, Kentenich C, Janni W, et al: Lack of effect of adjuvant chemotherapy on the elimination of single dormant tumor cells in the bone marrow of high-risk breast cancer patients. J Clin Oncol 18: 80-86, 2000.
    15. Muller V, Stahman N, Riethdorf S, et al: Circulating tumor cells in breast cancer: Correlation to bone marrow micrometastases. Heterogenous response to systemic therapy and low proliferative activity. Clin Cancer Res 11: 3678-3685, 2005.
    16. Cristofanilli M, Budd T, Ellis M, et al: Circulating tumor cells, disease progression, and survival in metastatic breast cancer. N Engl J Med 351: 781-791, 2004.
    17. Shewchuk, L, Hassell A, Wisely B, et al: Binding mode of the 4-anilinoquinazoline class of protein kinase inhibitor: X-ray crystallographic studies of 4-anilinoquinazolines bound to cyclin-dependent kinase 2 and p38 kinase. J Med Chem 43: 133-138, 2000.
    18. Yarden Y and Sliwkowski M.X: Untangling the ErbB signaling network. Nat Rev Mol Cell Biol 2: 127-137, 2001.
    19. Burgess, A.W, Cho H.S, Eigenbrot C, et al: An open-and-shut case? Recent insights into the activation of EGF/ErbB receptors. Mol Cell 12: 541-552, 2003.
    20. Graus-Porta D, Beerli R.R, Daly J.M, et al: ErbB2, the preferred heterodimerization partner of all ErbB receptors, is a mediator of lateral signaling. EMBO J 16: 1647-1655, 1997.
    21. Hendriks B.S, Opresko L.K, Wiley H.S, et al: Quantitative analysis of HER2-mediated effects on HER2 and epidermal growth factor receptor endocytosis. J Biol Chem 278: 23343-23351, 2003.
    22. Allred D.C, Mohsin S.K and Fuqua S.A.W. Histological and biological evolution of human premalignant breast disease. Endocr Rel Cancer 8: 47-61, 2001.
    23. Slamon DJ, Clark GM, Wong SG, et al: Human breast cancer: Correlation of relapse and survival with amplification of the HER-2/neu oncogene. Science 235: 177-182, 1987.
    24. Rusnak DW, Lackey K, Affleck K, et al: The effects of the novel, reversible epidermal growth factor recfeptor/ErbB2-tyrosine kinase inhibitor, GW572016, on the growth of human normal and tumor-derived cell lines in vitro and in vivo. Mol Cancer Ther 1: 85-94, 2001.
    25. Xia W, Mullin RJ, Keith BR, et al: Antitumor activity of GW 572016: A dual tyrosine kinase inhibitor blocks EGF activation of EGFR/erbB2 and downstream Erk1/2 and AKT pathways. Oncogene 21: 6255-6263, 2002.
    26. Zhou H, Ruth D, McCall W, et al: Radiosensitization of human breast cancer cells by lapatinib, a dual EGFR/HER2 tyrosine kinase inhibitor. Proc Am Assoc Cancer Res 2003 Abstract 4459.
    27. Lapatinib Investigator’s Brochure v.07, 06 April 2007, RM2000/00481/06
    28. Gomez HL, Chavez MA, Doval DC et al: Results from a phase II randomized study of lapatinib as first-line treatment for patients with ErbB2-amplified advanced or metastatic breast cancer. SABCS 2006 Abstract 1090.
    29. Burris H.A, Taylor C, Jones S, et al: A phase I study of lapatinib in patients with solid tumors. Proc Am Soc Clin Oncol 2003 Abstract 994.
    30. Spector N, Raefsky E, Hurwitz H, et al: Safety, clinical efficacy, and biologic assessments from EGF10004: A randomized phase IB study of lapatinib for patients with metastatic carcinomas over-expressing EGFR or ErbB2. Proc Am Soc Clin Oncol 2003 Abstract 772.
    31. Bacus S.S, Beresford P.J, Yarden Y, et al: The use of predicting factors and surrogate markers in patients’ cancer biopsies treated with targeted antibodies to erbB receptors and erbB tyrosine kinase inhibitors. Proc Am Soc Clin Oncol 2003 Abstract 3408
    32. Morris C. The role of EGFR-directed therapy in the treatment of breast cancer. Breast Cancer Res Treat 75: 51-55, 2002.
    33. Bozionellou V, Mavroudis D, Perraki M, et al: Trastuzumab administration can effectively target chemotherapy-resistant cytokeratin-19 messenger RNA-positive tumor cells in the peripheral blood and bone marrow of patients with breast cancer. Clin Cancer Res 10: 8185-8194, 2004.
    34. Brandt B, Roetger A, Heidl S, et al: Isolation of blood-bone epithelium-derived c-erb-B2 oncoprotein-positive clustered cells from the peripheral blood of breast cancer patients. Int J Cancer 76: 824-828, 1998.
    35. Braun S, Schlimok G, Heumos I, et al: ErbB2 overexpression on occult metastatic cells in bone marrow predicts poor clinical outcome of stage I-III breast cancer patients. Cancer Res 61: 1890-1895, 2001.
    36. Leitzel K, Lieu B, Curley E, et al : detection of cancer cells in peripheral blood of breast cancer patients using reverse transcription-polymerase chain reaction for epidermal growth factor receptor. Clin Cancer Res 4: 3037-3043, 1998.
    37. Kallergi G, Agelaki S, Kalykaki A, et al: EGFR, pAkt and pPI-3 kinase are co-expressed in circulating tumor cells of breast cancer patients. AACR 2007, abstr 5363.
    38. Konecny GE, Pegram MD, Venkatesan N, et al: Activity of dual kinase inhibitor lapatinib (GW572016) against HER-2 overexpressing and trastuzumab-treated breast cancer cell. Cancer Res 66: 1630, 2006.
    39. Mocellin S, Keilholz U, Rossi R, Nitti D. Trends in Molecular Med 2006.

**Appendix A World Medical Association Declaration of Helsinki**

Recommendations guiding physicians in biomedical research involving human subjects

Adopted by the 18th World Medical Assembly

Helsinki, Finland, June 1964

and amended by the

29th World Medical Assembly

Tokyo, Japan, October 1975

35th World Medical Assembly

Venice, Italy, October 1983

41st World Medical Assembly

Hong Kong, September 1989

and the

48th General Assembly

Somerset West, Republic of South Africa, October 1996

**INTRODUCTION**

It is the mission of the physician to safeguard the health of the people. His or her knowledge and conscience are dedicated to the fulfilment of this mission.

The Declaration of Geneva of the World Medical Association binds the physician with the words, “The health of my subject will be my first consideration,” and the International Code of Medical Ethics declares that, “A physician shall act only in the subject’s interest when providing medical care which might have the effect of weakening the physical and mental condition of the subject.”

The purpose of biomedical research involving human subjects must be to improve diagnostic, therapeutic and prophylactic procedures and the understanding of the etiology and pathogenesis of disease.

In current medical practice most diagnostic, therapeutic or prophylactic procedures involve hazards. This applies especially to biomedical research.

Medical progress is based on research which ultimately must rest in part on experimentation involving human subjects.

In the field of biomedical research a fundamental distinction must be recognized between medical research in which the aim is essentially diagnostic or therapeutic for a subject, and medical research, the essential object of which is purely scientific and without implying direct diagnostic or therapeutic value to the person subjected to the research.

Special caution must be exercised in the conduct of research which may affect the environment, and the welfare of animals used for research must be respected

Because it is essential that the results of laboratory experiments be applied to human beings to further scientific knowledge and to help suffering humanity, the World Medical Association has prepared the following recommendations as a guide to every physician in biomedical research involving human subjects. They should be kept under review in the future. It must be stressed that the standards as drafted are only a guide to physicians all over the world. Physicians are not relieved from criminal, civil and ethical responsibilities under the laws of their own countries.

**I. BASIC PRINCIPLES**

1. Biomedical research involving human subjects must conform to generally accepted scientific principles and should be based on adequately performed laboratory and animal experimentation and on a thorough knowledge of the scientific literature.

2. The design and performance of each experimental procedure involving human subjects should be clearly formulated in an experimental protocol which should be transmitted for consideration, comment and guidance to a specially appointed committee independent of the investigator and the sponsor provided that this independent committee is in conformity with the laws and regulations of the country in which the research experiment is performed.

3. Biomedical research involving human subjects should be conducted only by scientifically qualified persons and under the supervision of a clinically competent medical person. The responsibility for the human subject must always rest with a medically qualified person and never rest on the subject of research, even though the subject has given his or her consent.

4. Biomedical research involving human subjects cannot legitimately be carried out unless the importance of the objective is in proportion to the inherent risk to the subject.

5. Every biomedical research project involving human subjects should be preceded by careful assessment of predictable risks in comparison with foreseeable benefits to the subject or to others. Concern for the interests of the subject must always prevail over the interests of science and society.

6. The right of the research subject to safeguard his or her integrity must always be respected. Every precaution should be taken to respect the privacy of the subject and to minimize the impact of the study on the subject’s physical and mental integrity and on the personality of the subject.

7. Physicians should abstain from engaging in research projects involving human subjects unless they are satisfied that the hazards involved are believed to be predictable. Physicians should cease any investigation if the hazards are found to outweigh the potential benefits.

8. In publication of the results of his or her research, the physician is obliged to preserve the accuracy of the results. Reports of experimentation not in accordance with the principles laid down in this Declaration should not be accepted for publication.

9. In any research on human beings, each potential subject must be adequately informed of the aims, methods, anticipated benefits and potential hazards of the study and the discomfort it may entail. He or she should be informed that he or she is at liberty to abstain from participation in the study and that he or she is free to withdraw his or her consent to participation at any time. The physician should then obtain the subject’s freely-given informed consent, preferably in writing.

10. When obtaining informed consent for the research project the physician should be particularly cautious if the subject is in a dependent relationship to him or her or may consent under duress. In that case the informed consent should be obtained by a physician who is not engaged in the investigation and who is completely independent of this official relationship.

11. In case of legal incompetence, informed consent should be obtained from the legal guardian in accordance with national legislation. Where physical or mental incapacity makes it impossible to obtain informed consent, or when the subject is a minor, permission from the responsible relative replaces that of the subject in accordance with national legislation. Whenever the minor child is in fact able to give a consent, the minor’s consent must be obtained in addition to the consent of the minor’s legal guardian.

12. The research protocol should always contain a statement of the ethical considerations involved and should indicate that the principles enunciated in the present Declaration are complied with.

**II.MEDICAL RESEARCH COMBINED WITH PROFESSIONAL CARE (Clinical research)**

1. In the treatment of the sick person, the physician must be free to use a new diagnostic and therapeutic measure, if in his or her judgement it offers hope of saving life, re-establishing health or alleviating suffering.

2. The potential benefits, hazards and discomfort of a new method should be weighed against the advantages of the best current diagnostic and therapeutic methods.

3. In any medical study, every subject - including those of a control group, if any - should be assured of the best proven diagnostic and therapeutic method. This does not exclude the use of inert placebo in studies where no proven diagnostic or therapeutic method exists.

4. The refusal of the subject to participate in a study must never interfere with the physician–subject relationship.

5. If the physician considers it essential not to obtain informed consent, the specific reasons for this proposal should be stated in the experimental protocol for transmission to the independent committee (I, 2).

6. The Physician can combine medical research with professional care, the objective being the acquisition of new medical knowledge, only to the extent that medical research is justified by its potential diagnostic or therapeutic value for the subject.

**III.NON-THERAPEUTIC BIOMEDICAL RESEARCH INVOLVING HUMAN SUBJECTS (Non-clinical biomedical research)**

1. In the purely scientific application of medical research carried out on a human being, it is the duty of the physician to remain the protector of the life and health of that person on whom biomedical research is being carried out.

2. The subjects should be volunteers - either healthy persons or subjects for whom the experimental design is not related to the subject’s illness.

3. The investigator or the investigating team should discontinue the research if in his/her or their judgement it may, if continued, be harmful to the individual. In research on man, the interest of science and society should never take precedence over considerations related to the well being of the patient.

**Appendix B**

Sample written informed consent form

You are being asked to take part in a research study. Before you decide it is important for you to understand why the research is being done, what it will involve and the possible benefits, risks and discomforts. Please take time to read the following information carefully and discuss it with your family doctor, if you wish.

**What is the background and purpose of the study?**

Lapatinib is a new drug that has shown promising activity against breast cancer cancers. Lapatinib is a new treatment that has been shown to slow or stop growth in tumors. Lapatinib works differently from the way chemotherapy drugs work.

This study is being carried out to see if lapatinib is effective in eliminating tumor cells circulating in the blood of patients with breast cancer. Approximately 20 other patients like you will take part.

**Do I have to take part?**

It is up to you to decide whether or not to take part. If you do decide to take part you will be given this written informed consent to sign. If you decide to take part you are still free to withdraw at any time and without giving a reason. This will not affect the standard of care you receive.

Likewise, it may be decided by the Study Doctor that continuing participation in the study is no longer in your best interests and you will be withdrawn.

If you do not want to take part in the study there are alternative treatment options, which are available to treat your pancreatic cancer. Your Study Doctor can explain the treatments that are available to you if you choose not to take part.

**What will happen to me if I take part?**

The study involves approximately 6 visits over a period of approximately 24 weeks.

You will be given a copy of the study plan indicating when and what tests will be conducted at weekly intervals.

You will receive the study medication of lapatinib.

The study medication must be taken as follows:

**Lapatinib -** You will take the lapatinib tablet once a day, every day about the same time. You should take lapatinib on an empty stomach (either 1 hour before or 1 hour after meals). If you forget to take a dose, take the last missed dose as soon as you remember, as long as it is at least 12 hours before the next dose is due. If it is less than 12 hours to the next dose, do not take the dose you have missed.

**What do I have to do?**

You must be willing to attend all the scheduled visits, and to complete a series of brief questions as described by tour treating doctor at the nominated visits. It is also important that you take the study medication as directed. Any left over study medication that you do not take, and the container even if it is empty, must be returned at each of your visits. It is also important that you tell the medical staff about any other medication you are taking before and during the study.

You must not be pregnant or breast-feeding and you must not become pregnant during the study. You should use acceptable methods of birth control (i.e., birth control pills, condoms, approved contraceptive implant, intrauterine device, or tubal ligation) throughout the study to prevent pregnancy. Your Study Doctor must be told immediately if pregnancy occurs.

**What are the possible side effects, risks and discomforts of taking part?**

The study medication may cause some side effects. These are usually mild to moderate. Do not be alarmed by the list of side effects. You may experience none, some or all of those listed below.

Contact your Study Doctor promptly if any of the following happens to you, as you may need further examinations or treatment: fever, diarrhea or serious breathlessness. Very common side effects (more than 10 of every 100 patients are likely to have them) are: rash, diarrhea, nausea, vomiting, fatigue, anorexia Skin reactions such as acne-like rash, sometimes itchy with dry skin; Mild to moderate diarrhea; Nausea (feeling sick).

Take special care with lapatinib. If you get very breathless, or your breathlessness suddenly gets worse, possibly with a cough or fever, tell your doctor straight away. Some patients taking lapatinib get cardiac or pulmonary toxicity. These side effects are uncommon (less than 1 in every 100 patients).

There may be risks involved in taking this medication that have not been identified in the studies done so far. There is always a risk involved in taking a new medication but every precaution will be taken and you are encouraged to report anything that is troubling you. The taking of a blood sample may cause some pain, bruising, light headedness, and on rare occasions, infection.

You understand you will have to notify your Study Doctor immediately of any unusual side effects.

**What are the possible benefits of taking part?**

It is hoped that the lapatinib treatment will help you. However this cannot be guaranteed. The information we get from this study may help us to treat future subjects with breast cancer better.

**What if new information becomes available?**

If any new information on the medications becomes available which may influence your decision to continue in the study you will be told.

**Will the information collected be confidential?**

If you consent to take part in the study, any of your medical records will be directly inspected by the company sponsoring the study, contractors working on behalf of the sponsoring company and may also be inspected by the Regulatory Authorities and/or the Independent Ethics Committee to check that the study is being carried out correctly. By signing the written informed consent form you are giving permission for this to be done.

The information collected during the study will be stored in a computer but your name will not be.

Data collected during the study, will be submitted to the company sponsoring the study and contractors working on behalf of the sponsor, and may be submitted to the Regulatory Authorities outside the European Economic Area for the purpose of safety and efficacy evaluation and approval to market the study medication.

**What are the costs of participating?**

The expected cost of participation will be discussed with the Study Doctor at the point of Study Enrolment.

If you are caused any injury by your participation in the study, the company will compensate you in accordance with the law of Greece. The company is insured in this respect.

**Who should I contact if I need more information or help?**

In case of study-related injury or whenever you have questions about the study, or your study medication, please contact:

Dr (name) Nurse (name) Phone No. Phone No.

I have read and understand the patient information sheet which details how my tumor, peripheral blood, and plasma samples, will be used and stored, and how the information from the tests will be used, and I understand that consent that I give now is for the lifetime of the sample and that refusal to consent to the research use of biological materials will in no way affect my clinical care.

The peripheral blood, plasma and tumor samples collected in this trial, the results of any testing, and any patents, diagnostic tests, drugs, and biological products developed directly or indirectly as a result of this trial, as well as any information derived directly or indirectly from those samples, are the sole property of the GSK (and its successors, licensees, and assigns). I have no right to this property or to any share of any profits that may be earned directly or indirectly as a result of this trial.

I agree to a coded use of my biological materials for future non-genetic studies.
