# Supplementary material for: Efficacy of Lapatinib in Therapy-Resistant HER2-Positive Circulating Tumor Cells in Metastatic Breast Cancer
Source: PLoS One. 2015 Jun 17;10(6):e0123683. doi: 10.1371/journal.pone.0123683 (PMC4471111; doi:10.1371/journal.pone.0123683)
Supplement: S1 Table — (DOC) [file pone.0123683.s003.doc]

**Table S1: All grades toxicity observed in all patients and all cycles (n=22).**

|  | **Grade I** | | **Grade II** | | **Grade III** | | **Grade IV** | |
| --- | --- | --- | --- | --- | --- | --- | --- | --- |
|  | **No of pts** | **%** | **No of pts** | **%** | **No of pts** | **%** | **No of pts** | **%** |
| **Diarrhoea** | 8 | 36.4 | 3 | 13.6 | 1 | 4.5 | - | **-** |
| **Fatigue** | 8 | 36.4 | 2 | 9.0 | - | - | - | **-** |
| **Rash** | 5 | 22.7 | 2 | 9.0 | - | - | - | **-** |
| **Mucositis** | 3 | 13.0 | 1 | 4.5 | 1 | 13.6 | - | **-** |
| **Νausea/vomiting** | 5 | 22.7 | 1 | 4.5 | - | - | - | **-** |
| **Paronychia** | 4 | 18.2 | - | - | - | - | - | **-** |
| **Epistaxis** | 4 | 18.2 | - | - | - | - | - | **-** |
